# Supplementary material for: Initial presentation of early rheumatoid arthritis
Source: PLoS One. 2023 Jul 6;18(7):e0287707. doi: 10.1371/journal.pone.0287707 (PMC10325069; doi:10.1371/journal.pone.0287707)
Supplement: S1 Table — (DOCX) [file pone.0287707.s001.docx]

| Joint | All | Seropositive | Seronegative |  |
| --- | --- | --- | --- | --- |
| Right PIPs  PIP1  PIP2  PIP3  PIP4  PIP5 | 9.5 %  25.7 %  31.5 %  17.5 %  14.2 % | 8.0 %  22.3 %  27.5 %  14.3 %  12.0 % | 15.6 %  38.9 %  47.2 %  30.1 %  21.6 % |  |
| Left PIPs  PIP1  PIP2  PIP3  PIP4  PIP5 | 8.2 %  23.4 %  25.4 %  14.7 %  12.4 % | 6.5 %  20.5 %  21.1 %  10.5%  9.8 % | 15.1 %  34.9 %  42.0 %  31.0 %  21.6 % |  |
| Right MCPs  MCP1  MCP2  MCP3  MCP4  MCP5 | 17.1 %  34.9 %  28.4 %  9.5 %  12.0 % | 14.6 %  30.3 %  23.1 %  5.6 %  8.2 % | 27.0 %  52.8 %  48.9 %  24.7 %  26.7 % |  |
| Left MCPs  MCP1  MCP2  MCP3  MCP4  MCP5 | 13.3 %  28.7 %  23.9 %  9.7 %  9.9 % | 10.6 %  24.7 %  18.7 %  6.1 %  6.1 % | 23.9 %  44.0 %  44.3 %  24.1 %  24.7 % |  |
| Wrists  Right  Left | 43.6 %  43.5 % | 38.9 %  38.1 % | 65.1 %  61.4 % |  |
| Elbows  Right  Left | 6.8 %  5.9 % | 5.6 %  4.7 % | 11.4 %  10.5 % |  |
| Shoulders  Right  Left | 9.6 %  7.9 % | 9.0 %  7.4 % | 11.9 %  9.9 % |  |
| Hips  Right  Left | 1.9 %  2.2 % | 1.1 %  1.3 % | 4.8 %  5.7 % |  |
| Knees  Right  Left | 20.8 %  17.2 % | 18.6 %  15.2 % | 29.3 %  24.7 % |  |
| Ankles  Right  Left | 13.5 %  12.8 % | 10.5 %  10.1 % | 25.0 %  23.0 % |  |
| Right MTPS  MTP1  MTP2  MTP3  MTP4  MTP5 | 10.3 %  24.8 %  28.0 %  22.6 %  15.4 % | 8.9 %  23.6 %  27.2 %  21.4 %  15.2 % | 15.9 %  29.5 %  31.2 %  27.0 %  16.5 % |  |
| Left MTPS  MTP1  MTP2  MTP3  MTP4  MTP5 | 11.0 %  25.4 %  27.6 %  21.6 %  15.0 % | 9.7 %  24.1 %  26.6 %  19.8 %  14.5 % | 16.2 %  30.7 %  31.2 %  28.4 %  17.0 % |  |
| Temporomandibular joints  Right  Left | 0.1 %  0.2 % | 0.1 %  0.2 % | 0 %  0 % |  |

**S1 Table. Proportions of patients with a swollen joint from each of the sites in SJC46.**
