# Supplementary material for: Initial presentation of early rheumatoid arthritis
Source: PLoS One. 2023 Jul 6;18(7):e0287707. doi: 10.1371/journal.pone.0287707 (PMC10325069; doi:10.1371/journal.pone.0287707)
Supplement: S2 Table — (DOCX) [file pone.0287707.s002.docx]

| Variable | Available data for patients with a diagnostic delay of 0-3 months n, (%) | 0-3 months | Available data for patients with a diagnostic delay of 3-6 months n, (%) | 3-6 months | Available data for patients with a diagnostic delay of >6 months n, (%) | >6 months | p-value |
| --- | --- | --- | --- | --- | --- | --- | --- |
| n |  | 490 |  | 310 |  | 445 |  |
| Females n, % | 490 (100 %) | 319 (65 %) | 310 (100 %) | 196 (63 %) | 445 (100 %) | 295 (66 %) |  |
| Mean (SD) Age | 490 (100 %) | 61 (15) | 310 (100 %) | 58 (16) | 445 (100 %) | 56 (16) | <0.001 |
| Serology n, %  Seropositive  ACPA-positive  RF-positive | 490 (100 %)  489 (100 %)  472 (96 %) | 381 (78 %)  343 (70 %)  320 (68 %) | 310 (100 %)  309 (100 %)  302 (97 %) | 238 (77 %) 216 (70 %)  201 (67 %) | 445 (100 %)  444 (100 %)  437 (98 %) | 367 (82 %)  342 (77 %)  309 (71 %) | 0.115  0.045  0.324 |
| Median (IQR) SJC46 | 465 (95 %) | 6 (3, 11) | 301 (97 %) | 6 (3, 11) | 422 (95 %) | 6 (3, 10) | 0.665 |
| Median (IQR) TJC46 | 465 (95 %) | 7 (3, 13) | 301 (97 %) | 6 (3, 12) | 422 (95 %) | 5 (3, 10) | 0.059 |
| Proportions of patients with different SJC46 n, %  0-2  3-5  ≥6 |  | 114 (24 %)  101 (22 %)  249 (53 %) |  | 53 (18 %)  74 (25 %)  174 (58 %) |  | 93 (22 %)  117 (28 %)  212 (50 %) | 0.385 |
| Proportions of patients with different TJC46 n, %  0-2  3-5  ≥6 |  | 97 (21 %)  101 (22 %)  267 (57 %) |  | 52 (17 %)  85 (28 %)  164 (54 %) |  | 98 (23 %)  121 (29 %)  203 (48 %) | 0.184 |
| Median (IQR) CRP | 471 (96 %) | 11 (4, 30) | 300 (97 %) | 8 (3, 24) | 419 (94 %) | 7 (3, 19) | 0.008 |
| Median (IQR) ESR | 424 (87 %) | 27 (12, 44) | 271 (87 %) | 20 (10, 36) | 386 (87 %) | 18 (9, 34) | <0.001 |
| Proportions of patients with normal CRP or ESR n, %  CRP  ESR |  | 216 (46 %)  232 (55 %) |  | 164 (55 %)  165 (61 %) |  | 247 (59 %)  256 (66 %) | 0.008  0.046 |
| Median (IQR) DAS28 | 454 (93 %) | 4.1 (3.3, 5.1) | 292 (94 %) | 4.0 (3.2, 4.8) | 405 (91 %) | 3.8 (3.1, 4.5) | 0.016 |
| Median (IQR) Dr.global | 428 (87 %) | 40 (27, 58) | 272 (88 %) | 40 (30, 55) | 388 (87 %) | 40 (25, 60) | 0.893 |
| Median (IQR) PROs  Pain  Fatigue  PGA | 441 (90 %)  412 (84 %)  442 (90 %) | 62 (35, 78)  50 (18, 71)  51 (29, 70) | 291 (94 %)  279 (90 %)  291 (94 %) | 52 (30, 72)  48 (22, 70)  50 (26, 67) | 394 (89 %)  365 (82 %)  396 (89 %) | 50 (26, 70)  43 (13, 69)  46 (22, 62) | <0.001  0.527  0.019 |
| Median (IQR) HAQ | 393 (80 %) | 1.10 (0.50, 1.60) | 258 (83 %) | 0.9 (0.50, 1.40) | 356 (80 %) | 0.75 (0.38, 1.2) | 0.002 |

**S2 Table. Clinical data of patients with incident RA by diagnostic delay.**
