# Supplementary material for: Initial presentation of early rheumatoid arthritis
Source: PLoS One. 2023 Jul 6;18(7):e0287707. doi: 10.1371/journal.pone.0287707 (PMC10325069; doi:10.1371/journal.pone.0287707)
Supplement: Attachment — Submitted filename: PONE-D-22-32344_R1.pdf [file pone.0287707.s004.pdf]

# PLOS ONE

## Initial presentation of early rheumatoid arthritis

--Manuscript Draft--

| | |
|---|---|
| **Manuscript Number:** | PONE-D-22-32344R1 |
| **Article Type:** | Research Article |
| **Full Title:** | Initial presentation of early rheumatoid arthritis |
| **Short Title:** | - |
| **Corresponding Author:** | Lauri Weman, M.D<br>University of Eastern Finland Faculty of Health Sciences: Ita-Suomen yliopisto Terveystieteiden tiedekunta<br>Kuopio, FINLAND |
| **Keywords:** | Rheumatoid arthritis; ACPA; diagnostic delay |
| **Abstract:** | Objectives:<br>To study the joint distribution and clinical picture of rheumatoid arthritis (RA) at the initial presentation in anti-citrullinated protein antibody (ACPA)-positive and negative patients and the effect of diagnostic delay on the clinical picture.<br><br>Methods<br><br>Data of patients who received reimbursement for DMARDs for newly diagnosed RA in 1/2019 to 9/2021 were extracted from the national databases. Swollen and tender joint counts, presence of symmetrical swelling, other disease activity measures, and patient reported outcomes (PROs) were compared in ACPA positive and negative patients. Regression analyses were applied to compare clinical variables in patients with duration of symptoms of <3, 3-6, and >6 months, adjusted for age, sex, and ACPA-positivity.<br><br>Results<br><br>Data of 1801 ACPA-tested patients were included. Symmetrical swelling was present in 75% of patients. ACPA negative versus positive patients had higher value for all disease activity measures and PROs including median swollen joint count (SJC46 9 versus 5) and DAS28 (4.5 versus 3.7), (p<0.001). Patients diagnosed in <3 months had higher median pain VAS (62 versus 52 and 50, p<0.001) and HAQ (1.1 versus 0.9 and 0.75, p=0.003) compared to those with a duration of symptoms of 3-6 and >6 months. Patients diagnosed >6 months were ACPA positive more frequently (76% versus 69% in other groups, p<0.001).<br><br>Conclusion<br><br>Incident RA presents mainly as symmetric arthritis. ACPA-negative patients have higher disease burden at the initial presentation. Patients experiencing more severe pain and decreased functional ability are diagnosed earlier, regardless of ACPA status. |
| **Order of Authors:** | Lauri Weman, MD |
| | Henri Salo, MSc |
| | Laura Kuusalo, MD, PHD |
| | Johanna Huhtakangas, MD, PHD |
| | Paula Vähäsalo, MD, PHD |
| | Maria Backström, MD, PHD |
| | Johanna Kärki, MD |
| | Tuulikki Sokka-Isler, MD, PHD |
| **Response to Reviewers:** | Dear Sadiq Umar |

Powered by Editorial Manager® and ProduXion Manager® from Aries Systems Corporation

| | |
|---|---|
| | We have now made all the corrections you suggested to us for the manuscript called "initial presentation of rheumatoid arthritis". I hope this is now acceptable for your paper.<br><br>B.R Dr Weman |
| Additional Information: | |
| **Question** | **Response** |
| **Financial Disclosure**<br><br>Enter a financial disclosure statement that describes the sources of funding for the work included in this submission. Review the submission guidelines for detailed requirements. View published research articles from *PLOS ONE* for specific examples.<br><br>This statement is required for submission and **will appear in the published article** if the submission is accepted. Please make sure it is accurate.<br><br>**Unfunded studies**<br>Enter: *The author(s) received no specific funding for this work.*<br><br>**Funded studies**<br>Enter a statement with the following details:<br>• Initials of the authors who received each award<br>• Grant numbers awarded to each author<br>• The full name of each funder<br>• URL of each funder website<br>• Did the sponsors or funders play any role in the study design, data collection and analysis, decision to publish, or preparation of the manuscript?<br>• **NO** - Include this sentence at the end of your statement: *The funders had no role in study design, data collection and analysis, decision to publish, or preparation of the manuscript.*<br>• **YES** - Specify the role(s) played.<br><br>* typeset | We would like to thank The Finnish Psoriasis Association, The Finnish Society for Rheumatology, and Amgen. The author also acknowledges the Research Committee of the Kuopio University Hospital Catchment Area for the State Research Funding (project 5041730, Kuopio, Finland). |
| **Competing Interests**<br><br>Use the instructions below to enter a competing interest statement for this submission. On behalf of all authors, disclose any competing interests that | The authors have declared that no competing interests exist. |

Powered by Editorial Manager® and ProduXion Manager® from Aries Systems Corporation

| | |
|---|---|
| could be perceived to bias this work—acknowledging all financial support and any other relevant financial or non-financial competing interests.<br><br>This statement is required for submission and **will appear in the published article** if the submission is accepted. Please make sure it is accurate and that any funding sources listed in your Funding Information later in the submission form are also declared in your Financial Disclosure statement.<br><br>View published research articles from *PLOS ONE* for specific examples.<br><br>**NO authors have competing interests**<br><br>Enter: *The authors have declared that no competing interests exist.*<br><br>**Authors with competing interests**<br><br>Enter competing interest details beginning with this statement:<br><br>*I have read the journal's policy and the authors of this manuscript have the following competing interests: [insert competing interests here]*<br><br>* typeset | |
| **Ethics Statement**<br><br>Enter an ethics statement for this submission. This statement is required if the study involved:<br><br>• Human participants<br>• Human specimens or tissue<br>• Vertebrate animals or cephalopods<br>• Vertebrate embryos or tissues<br>• Field research<br><br>Write "N/A" if the submission does not require an ethics statement. | This study was conducted as a register-based study using data from the Finnish Rheumatology Quality Register, which is kept by the Finnish Institute for Health and Welfare (THL) and granted approval for the study. In a register-based study, patient consent was not required. |

| | |
|---|---|
| General guidance is provided below. Consult the submission guidelines for detailed instructions. **Make sure that all information entered here is included in the Methods section of the manuscript.**<br><br>**Format for specific study types**<br><br>**Human Subject Research (involving human participants and/or tissue)**<br>• Give the name of the institutional review board or ethics committee that approved the study<br>• Include the approval number and/or a statement indicating approval of this research<br>• Indicate the form of consent obtained (written/oral) or the reason that consent was not obtained (e.g. the data were analyzed anonymously)<br><br>**Animal Research (involving vertebrate animals, embryos or tissues)**<br>• Provide the name of the Institutional Animal Care and Use Committee (IACUC) or other relevant ethics board that reviewed the study protocol, and indicate whether they approved this research or granted a formal waiver of ethical approval<br>• Include an approval number if one was obtained<br>• If the study involved *non-human primates*, add *additional details* about animal welfare and steps taken to ameliorate suffering<br>• If anesthesia, euthanasia, or any kind of animal sacrifice is part of the study, include briefly which substances and/or methods were applied<br><br>**Field Research**<br><br>Include the following details if this study involves the collection of plant, animal, or other materials from a natural setting:<br>• Field permit number<br>• Name of the institution or relevant body that granted permission | |
| **Data Availability**<br><br>Authors are required to make all data | No - some restrictions will apply |

| | |
|---|---|
| underlying the findings described fully available, without restriction, and from the time of publication. PLOS allows rare exceptions to address legal and ethical concerns. See the PLOS Data Policy and FAQ for detailed information.<br><br>A Data Availability Statement describing where the data can be found is required at submission. Your answers to this question constitute the Data Availability Statement and **will be published in the article**, if accepted.<br><br>**Important:** Stating 'data available on request from the author' is not sufficient. If your data are only available upon request, select 'No' for the first question and explain your exceptional situation in the text box.<br><br>Do the authors confirm that all data underlying the findings described in their manuscript are fully available without restriction? | |
| **Describe where the data may be found in full sentences. If you are copying our sample text, replace any instances of XXX with the appropriate details.**<br><br>• If the data are **held or will be held in a public repository**, include URLs, accession numbers or DOIs. If this information will only be available after acceptance, indicate this by ticking the box below. For example: *All XXX files are available from the XXX database (accession number(s) XXX, XXX.*).<br>• If the data are all contained **within the manuscript and/or Supporting Information files**, enter the following: *All relevant data are within the manuscript and its Supporting Information files.*<br>• If neither of these applies but you are able to provide **details of access elsewhere**, with or without limitations, please do so. For example:<br><br>*Data cannot be shared publicly because of [XXX]. Data are available from the* | The Quality Register data must be used strictly for the purposes of the Quality register, by the specialists nominated for this task, under the Finnish Institute for Health and Welfare, which is supervised by the Ministry of Social Affairs and Wealth. The Finnish Institute for Health and Welfare is the owner of the data.<br><br>Queries concerning the quality register data can be directed to the institute FinData, which is mandated to grant approval to use anonymized quality register data for research purposes.<br><br>E-mail: data@findata.fi |

| | |
|---|---|
| *XXX Institutional Data Access / Ethics Committee (contact via XXX) for researchers who meet the criteria for access to confidential data.*<br><br>*The data underlying the results presented in the study are available from (include the name of the third party and contact information or URL).*<br>• This text is appropriate if the data are owned by a third party and authors do not have permission to share the data.<br><br>* typeset | |
| Additional data availability information: | |

Powered by Editorial Manager® and ProduXion Manager® from Aries Systems Corporation

Manuscript

Click here to access/download;Manuscript;Revised_manuscript.docx



Central Finland Health Care District, UEF

# Initial presentation of early rheumatoid arthritis

Lauri Weman[1]*, Henri Salo [2], Laura Kuusalo [3], Johanna Huhtakangas [4], Johanna Kärki [5], Paula Vähäsalo [6], Maria Backström [7]

Tuulikki Sokka-Isler [8]

[1] Faculty of Health Sciences, University of Eastern Finland, Kuopio, Finland

[2] Finnish institute for health and welfare (THL), Helsinki, Finland

[3] Centre for Rheumatology and Clinical Immunology, Turku University Hospital, Turku, Finland

[4] Division of Rheumatology, Kuopio University Hospital, Kuopio, Finland

[5] Department of Children and Adolescents, Kanta-Häme Central Hospital, Hämeenlinna, Finland

[6] PEDEGO Research Unit, University of Oulu, Oulu, Finland

[7] Department of Pediatrics, Vaasa Central Hospital, Vaasa, Finland

[8] Department of Rheumatology, Central Finland Central Hospital, Jyväskylä, Finland

*Corresponding author

E-mail:lauriweman@gmail.com

This study received no external funding. None of the authors had any conflicts of interest.

# Abstract

*Objectives:* To study the joint distribution and clinical picture of rheumatoid arthritis (RA) at the initial presentation in seropositive (anti-citrullinated protein antibody (ACPA) and/or rheumatoid factor (RF) positive) and negative patients and the effect of duration of symptoms on the clinical picture.

*Methods:* Data of patients who received reimbursement for DMARDs for newly diagnosed RA in 1/2019 to 9/2021 were extracted from the national databases. Joint counts, presence of symmetrical swelling, other disease activity measures, and patient reported outcomes (PROs) were compared in seropositive and negative patients. Regression analyses were applied to compare clinical variables in patients with duration of symptoms of <3, 3-6, and >6 months, adjusted for age, sex, and seropositivity.

*Results:* Data of 1816 ACPA and RF-tested patients were included. Symmetrical swelling was present in 75 % of patients. Seronegative *versus* positive patients had higher value for all disease activity measures and PROs including median swollen joint count (SJC46 10 *versus* 5) and DAS28 (4.7 *versus* 3.7), (p<0.001). Patients diagnosed in <3 months had higher median pain VAS (62 *versus* 52 and 50, p<0.001) and HAQ (1.1 *versus* 0.9 and 0.75, p=0.002) compared to those with a duration of symptoms of 3-6 and >6 months. Patients diagnosed >6 months were ACPA-positive more frequently (77 % *versus* 70 % in other groups, p=0.045).

*Conclusion:* Incident RA presents mainly as symmetric arthritis. Seronegative patients have higher disease burden at the initial presentation. Patients experiencing more severe pain and decreased functional ability are diagnosed earlier, regardless of ACPA- status.

## Background

Before the introduction of modern diagnostic tools, incident rheumatoid arthritis (RA) was recognized as symmetric polyarthritis. Symmetry was one criterion in the previous 1987 classification criteria for RA (1), which were developed to differentiate RA from other types of inflammatory arthritides. Symmetry of joint erosions has been observed in patients with early RA diagnosed in the early 1990's (2) and in patients with established RA in 1963-1996 (2,3). However, no studies have been conducted recently on the symmetry of joint involvement at the presentation of RA (4). Although not part of the current classification criteria (5), symmetry of arthritis is still being taught in textbooks and medical schools, as one of the hallmarks of incident RA. However, considering RA only in cases with symmetric polyarthritis might postpone the suspicion of early RA in primary health care.

In the 2010 ACR/EULAR classification criteria for RA, more emphasis has been placed on serology (5) without requirement for symmetry. With the 2010 criteria, early RA is now presented mainly in two forms with 6 criteria fulfilled, either seropositive RA, which can be oligoarthritis without elevated inflammatory markers, or seronegative RA, which is usually polyarthritis with elevated inflammatory markers. In fact, it is getting obvious that autoantibodies are the hallmark of RA and seronegative RA is a distinct entity (6) with different risk factors (7,8) and outcomes compared to seropositive RA (9). Our recent study indicated that seronegative RA turns out to be a spectrum of different conditions when observed for 10 years (10,11). Nonetheless, seronegative active arthritis requires early identification and tightly controlled treatment with Disease Modifying Antirheumatic Drugs (DMARDs) as it also causes significant treatable disease burden to patients (12).

First in the 1970's (13) and more widely since the 1990's, it has been recognized that RA should be identified and its treatment initiated as soon as possible to facilitate good outcomes (13,14). With multiple studies showing that early recognition is crucial, it has since been emphasized as an important approach for the management of RA (15). It has also been shown that treatment initiation within the first 12 weeks after the first symptoms might be the best window of opportunity in terms of treatment outcomes (16,17). Some studies suggest that the duration of symptoms has decreased during the current millennium (18). However, there are still notable delays in terms of treatment initiation. For example, only 31 % of patients with incident RA were examined by a rheumatologist

within 12 weeks of the first symptoms in the Netherlands in 1993-2006 (19). In the UK, patients with early RA visit their general practitioner 4 times on average before being referred to a specialist according to a report from 2008-2009 (20).

Therefore, our objective was to study the initial presentation of RA in a nationwide setting, with a focus on the serological status and symmetry of joint swelling, as well as the effect of duration of symptoms.

# Methods

**Setting.** Patients with incident RA are diagnosed and treated in rheumatology outpatient clinics in Finland. According to the national guidelines, DMARDs are started at the time of the diagnosis together with a medication reimbursement application, prepared by the rheumatologist. The reimbursement is granted by the Social Insurance Institution of Finland (KELA) for DMARDs. In addition to granting reimbursements for patients with chronic illnesses, such as RA, KELA also maintains a database of the individuals, containing the ICD code of the diagnosis, date of the reimbursement and basic demographic data of the individuals.

For the current study, patients who received their first reimbursement for DMARDs prescribed for the treatment of RA between 1/2019-9/2021 were identified in the KELA database. Clinical and demographic data were extracted from The Finnish Rheumatology Quality Register, using the individual identification code. To capture the data at the time of the diagnosis, clinical data were extracted from the most recent visit within 0 to 90 days prior to the date of the medication reimbursement; visits occurred between 23rd November 2018 and 30th August 2021. All patients were over 16 years old and could not be diagnosed as any other specific arthritis. The ACR/EULAR 2010 classification criteria were used to aid in diagnosing the patients (5).

**Variables.** Following variables were collected at the time of the diagnosis of RA and were available for analyses.

*Demographic variables*

- Age in years
- Sex; male subjects/female subjects
- Smoking status: current smoking; having ever smoked
- Employment status for patients under 65 years old; currently working, unemployed, disabled and not in work force such as home makers and students

*Disease characteristics*

- Duration of symptoms
- Fulfillment of ACR/EULAR 2010-criteria (5).

*Clinical variables*

- Swollen joint count (SJC) and Tender joint count (TJC) on 46 joint counts
- Distribution of swollen and tender joints on 46 joint counts
- Presence of symmetrical swelling in the MCPs, PIPs, wrists, MTPs, knees, ankles, and the elbows
- C-reactive protein (CRP), erythrocyte sedimentation rate (ESR) and DAS28 scores
- Investigator's global assessment of disease activity (Dr.global)
- Patient reported outcomes (PROs) such as pain, fatigue, and Patient Global Assessment (PGA)
- Self-reported functional capacity according to the Stanford Health Assessment Questionnaire (HAQ).

*The Initial Medication*

- The medications of interest were methotrexate (MTX), other conventional synthetic disease-modifying antirheumatic drugs (csDMARDs), such as hydroxychloroquine (HCQ) and sulfasalazine (SSZ), biological DMARDs (bDMARDs) and janus kinase (JAK) inhibitors, as well as systemic glucocorticoids (GC).

*Duration of symptoms* is the time between the first symptoms and the diagnosis of RA, recorded in months. At the initial visit, the patient was asked when he/she recognized the first symptoms of RA.

*ACPA- positivity* was defined by the laboratory reference values, which was ≥ 7 kU/l.

*RF-positivity* was defined by the laboratory reference values, being ≥ 15 IU/ml.

*Fulfilment of the ACR/EULAR 2010 criteria* was calculated by the examining physician, who counts the items in the ACR/EULAR 2010-criteria. If the total score adds up to 6 or more points, the ACR/EULAR 2010-criteria are met, when other reasons that can explain the condition are excluded (5).

*Joint counts.* The presence of swollen and tender joints was calculated using a 46 joint count including proximal interphalangeal (PIP), and metacarpophalangeal (MCP) joints, interphalangeal (IP) joints of the thumbs, wrists, metatarsophalangeal (MTP) joints, distal interphalangeal (DIP) joints of big toes, temporomandibular joints as well as ankle-, knee-, hip-, elbow- and shoulder joints.

*Symmetrical swelling* was analyzed from the MCPs, PIPs, wrists, MTPs, knees, ankles and the elbows, according to the 1987 criteria (1). A patient was considered to have symmetrical swelling if he/she had bilateral swelling in the same anatomical site. Symmetrical joint tenderness wasn't analyzed as it wasn't in the 1987 criteria (1).

*CRP and ESR* were determined according to the laboratory reference values for women and men in different age groups. A reference value of below 10 mg/l was normal for CRP. An elevated ESR included values of ≥ 20 mm/h for women younger than 50 years, ≥ 30 mm/h for women over 50 years and ≥42 mm/h for women over 85 years and ≥ 15 mm/h for men younger than 50 years, ≥20 mm/h for men over 50 years and ≥30 mm/h for men over 85 years.

*DAS28* was used to describe disease activity (21).

*Dr.global* included the physicians' assessment of rheumatic activity on the 0-100 mm Visual Analog Scale (VAS), where 0 equals no disease activity and 100 maximal disease activity.

*PROs* included the self-assessment of pain, fatigue, and PGA on the 0-100 mm VAS, where 0 equals no symptoms and 100 maximal discomfort.

*HAQ* was used to describe the functional capacity of patients. It is scored from 0 to 3, a score of <0.5 is a sign of good functional status. In this study, we used HAQ without ''aids and devices'' due to its better accuracy in clinical studies (22,23).

*The Initial Medications* were analyzed in following groups:

- MTX monotherapy
- MTX in combination with (an)other csDMARD(s)
- csDMARD monotherapy or a combination
- bDMARD with or without MTX
- JAK-inhibitor with or without MTX
- GC, regardless of DMARDs

Clinical and demographic variables were determined for all patients and for seropositive and negative patients. Comparisons were conducted between seropositive and negative patients as well as between only ACPA-positive and only RF-positive patients with clinical variables. The use of medication was determined only for seropositive and seronegative patients and their use was compared between these groups.

## Methods to study the effect of duration of symptoms on disease activity.

Three groups were formed according to the duration of symptoms before receiving the diagnosis: patients with a duration of symptoms of <3 months (group 1), patients with a duration of symptoms of 3-6 months (group 2) and patients with a duration of symptoms of over 6 months (group 3). SJC46, TJC46, serological status, CRP, ESR, DAS28, PROs, HAQ, and Dr.global were compared between these groups.

**Statistical methods.** Descriptive statistics were used with mean values with standard deviation (SD) and median values with interquartile ranges (IQR) depending on the way a value is distributed. Chi-square test was used to compare categorical variables. P=0.05 was set as a threshold for statistical significance. Regression models were used to compare clinical and demographic variables, ANOVA for continuous variables and logistic regression for dichotomous correlatives and for comparisons of median values transformed as median splits. Adjustments for age and sex were used in the comparisons of patients by ACPA-status. Variables were adjusted for seropositivity, age and sex in the comparisons of groups by duration of symptoms.

Analyses were conducted using the R Statistical language (version 4.2.1; R Core Team, 2022) on Ubuntu 20.04.5 LTS.

## Ethical issues.

This study was conducted as a register-based study using data from the Finnish Rheumatology Quality Register, which is kept by the Finnish Institute for Health and Welfare (THL), which granted the approval for the study. In a register-based study, patient consent was not required.

# Results

## Demographic variables

A total of 2017 patients with incident RA were identified in the database. ACPA and/or RF were available for 1816 (90 %) patients, which were included in the analyses. Of these, 1444 (80 %) were seropositive and 372 (20 %) were seronegative. A total of 5 (0.3 %) seropositive patients weren't tested for ACPA, as they were already RF-positive. Vice-versa, 83 (6 %) patients weren't tested for RF, as they already had a positive ACPA-result. The mean (SD) age was 59 (16) years for all patients. For seropositive and negative patients, the corresponding numbers were 59 (15) and 60 (16) ($p<0.001$). A total of 1034 (57 %) patients overall, 850 (59 %) seropositive patients and 184 (49 %) seronegative patients were under 65 years old at the initial visit ($p=0.002$) (Table 1).

A total of 19 % of all patients were current smokers and 55 % had ever smoked. The corresponding proportions were 21 % and 57 % for seropositive patients and 12 % and 46 % for seronegative patients ($p<0.001$ for both comparisons, adjusted for age and sex). In terms of employment status, a total of 69 % of all patients were employed, 20 % disabled, 8 % unemployed and 4 % weren't currently in work force. No major differences were found between seropositive and negative patients. (Table 1).

## Disease characteristics

The median (IQR) duration of symptoms before being diagnosed was 4 (2, 10) for all patients. For seropositive patients it was 5 (2, 10) and 4 (2, 8) for seronegative patients (p=0.030, adjusted for age and sex).

The proportions of patients fulfilling the ACR/EULAR 2010-criteria at the initial presentation were 88 % for all patients, 91 % for seropositive and 74 % for seronegative patients (p<0.001, adjusted for age and sex) (Table 1).

| **Variable** | **Available data for all patients** | **All** | **Available data for seropositive n, (%)** | **Seropositive** | **Available data for seronegative n, (%)** | **Seronegative** | **p-value** |
|---|---|---|---|---|---|---|---|
| **n** | 1816 (100 %) | 1816 | | 1444 (80 %) | | 372 (20 %) | |
| **Females, n (%)** | 1816 (100 %) | 1177 (65 %) | 1444 (100 %) | 947 (65 %) | 372 (100 %) | 230 (62 %) | |
| **Mean (SD) Age in years**<br>All | 1816 (100 %) | 59 (16) | 1444 (100 %) | 58 (16) | 372 (100 %) | 60 (16) | <0.001 |
| **Smoking status, n (%)**<br>Current smokers<br>Patients that have ever smoked | 1707 (94 %) | 321 (19 %)<br>942 (55 %) | 1363 (94 %) | 281 (21 %)<br>783 (57 %) | 344 (92 %) | 40 (12 %)<br>159 (46 %) | <0.001<br><0.001 |
| **Patients under 65 years old, n (%)** | | 1034 (57 %) | | 850 (59 %) | | 184 (49 %) | 0.002 |
| **Employment status for patients under 65 years old, n (%)**<br>Employed<br>Disabled<br>Unemployed<br>Not in work force | 733 (71 %) | 504 (69 %)<br>148 (20 %)<br>52 (8 %)<br>29 (4 %) | 601 (71 %) | 415 (69 %)<br>119 (20 %)<br>46 (8 %)<br>21 (3 %) | 132 (72 %) | 89 (67 %)<br>29 (22 %)<br>6 (5 %)<br>8 (6 %) | 0.273 |
| **Disease characteristics** | | | | | | | |

| | | | | | | | |
|---|---|---|---|---|---|---|---|
| **Median (IQR) duration of symptoms in months** | 1288 (71 %) | 4 (2, 10) | 1026 (71 %) | 5 (2, 10) | 262 (70 %) | 4 (2, 8) | 0.030 |
| **Proportions of patients fulfilling the ACR/EULAR 2010 criteria** | 1497 (82 %) | 1311 (88 %) | 1165 (81 %) | 1064 (91 %) | 332 (89 %) | 247 (74 %) | <0.001 |

**Table 1**. **Demographic data and disease characteristics of patients with RA at the initial presentation, by serological status.**

## Swollen- and tender joint counts

The median (IQR) SJC46 was 6 (3, 10) for all patients. For seropositive patients it was 5 (2, 9) and 10 (6, 15) for seronegative patients ($p<0.001$). The values for TJC46 were 6 (3, 11) for all patients and 5 (2, 10) and 10 (5, 18) for seropositive and negative patients, respectively ($p<0.001$, adjusted for age and sex) (Table 2).

For all patients, 23 % had two or less swollen joints at the initial presentation, 25 % had 3-5 and 52 % had ≥6 swollen joints on the 46 swollen joint count. The corresponding proportions were 26 %, 28 % and 46 % for seropositive patients and 9 %, 16 % and 75 % for seronegative patients ($p<0.001$, adjusted for age and sex) (Table 2).

A total of 23 % of all patients had two or less tender joints in the 46 tender joint count, 25 % had 3-5 and 52 % ≥6 tender joints. For seropositive patients the proportions were 26 %, 28 % and 46 % and 14 %, 16 % and 70 % for seronegative patients ($p<0.001$, adjusted for age and sex) (Table 2).

## Pattern and symmetry of joint involvement at the initial presentation

At the initial presentation, wrists were the most commonly affected joints, with 43.5 % of patients having a swollen left wrist and 43.6 % a swollen right wrist. Any joint from the right MCP's was swollen in 9.5 to 34.9 % of patients and in 9.7 to 28.7 % of patients from the left MCP's. The corresponding proportions were 9.5 to 31.5 % for the right PIP's and 8.2 to 25.4 % for the left PIP's and 10.3 to 28.0 % for the right and 11.0 to 27.6 % for the left MTPs. Knees were the most commonly swollen large joints (20.8 % for right and 17.2 % for the left). The corresponding proportions were 9.6 % and 7.9 % for shoulders, 13.5 % and 12.8 % for ankles, 6.8 % and 5.9 % for elbows and 1.9 % and 2.2 % for the hips (S1 Table).

At the initial presentation of RA, a total of 75 % of all patients had symmetrical swelling in any of the anatomical sites. In different anatomical sites, 35 % had symmetrical swelling in MCPs, 33 % in the PIPs, 34 % in wrists, 35 % in the MTPs, 12 % in the knees, 8 % in the ankles and 4 % in the elbows. A total of 71 % of seropositive patients had any symmetrical swelling, and the corresponding proportion was 91 % for seronegative patients (<0.001, adjusted for age and sex). In terms different anatomical sites, seronegative patients had more symmetrical swelling in all sites, with the biggest differences in the wrists compared to seropositive patients (28 % in seropositive and 56 % in

seronegative patients) (p<0.001, adjusted for age and sex) and the MCPs (29 % and 56 %) (p<0.001, adjusted for age and sex). A total of 34 % of seropositive patients had symmetrical swelling in the MTPs and 39 % of seronegative patients (p=0.097) (Table 2, Fig 1).

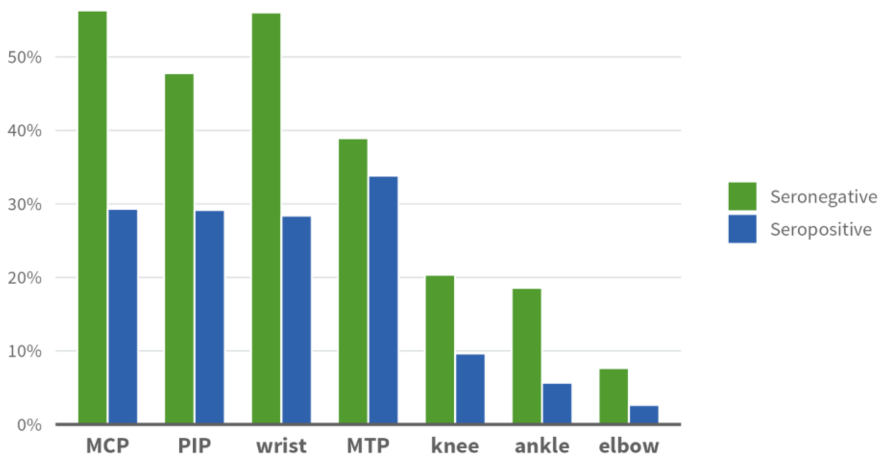


**Figure 1. Proportions of patients with symmetrical swelling by anatomical site in seropositive and seronegative patients, at the initial presentation of RA.**

## Disease activity measures

The median (IQR) CRP was 8 (3, 25) for all patients with incident RA, 7 (3, 21) for seropositive and 15 (5, 46) for seronegative patients (p<0.001). The median (IQR) ESR was 22 (10, 39) for all patients 20 (10, 37) for seropositive and 26 (12, 46) for seronegative patients (p=0.006, adjusted for age and sex).

A total of 53 % of all patients had normal CRP and 60 % normal ESR. For seropositive and negative patients, the proportions were 57 % and 35 % for CRP (p<0.001) and 63 % and 51 % for ESR, respectively (p<0.001, adjusted for age and sex) (Table 2).

At the initial presentation, the median (IQR) DAS28-scores were 3.9 (3.1, 4.7) for all patients, 3.7 (3.0, 4.4) for seropositive patients and 4.7 (4.0, 5.7) for seronegative patients (p<0.001, adjusted for age and sex) (Table 2).

For Dr.global, the median (IQR) value was 40 (26, 56) for all patients, 39 (25, 50) for seropositive patients and 50 (38, 69) for seronegative patients (p<0.001, adjusted for age and sex) (Table 2).

## Patient reported outcomes

For pain, the median (IQR) VAS-score was 54 (30, 75) for all patients, 51 (30, 74) for seropositive patients and 61 (39, 77) for seronegative patients (p<0.001, adjusted for age and sex). For fatigue, the corresponding numbers were 45 (18, 70), 40 (14, 69) and 52 (31, 73) (p<0.001, adjusted for age and sex) and for PGA they were 50 (25, 67), 48 (23, 64) and 53 (32, 70) (p<0.001, adjusted for age and sex) (Table 2).

The overall median (IQR) HAQ was 0.9 (0.5, 1.4) for all patients, 0.88 (0.38, 1.4) for seropositive patients and 1.1 (0.75, 1.5) for seronegative patients at the initial presentation (p<0.001, adjusted for age and sex) (Table 2).

## Initial medication

A total of 17 % of seropositive patients and 23 % of seronegative patients started with MTX monotherapy, 72% and 67% started MTX + csDMARD, 10% and 11% started csDMARD(s) only, while bDMARDs and JAK-inhibitors were started only for a few patients. GCs were started for 82 % of seropositive and 94 % of seronegative patients (Table 2).

**Commented [TS1]:** EI VOIDA SANOA PRESCRIBED KOSKA EI VOI TIETÄÄ MITÄ NIILLE OLI MÄÄRÄTTY, TIEDETÄÄN VAIN MITÄ OLIVAT OSTANEET.

| Variable | Available data for all patients n, (%) | All | Available data for seropositive patients n, (%) | Seropositive | Available data for seronegative patients n, (%) | Seronegative | p-value |
|---|---|---|---|---|---|---|---|
| **Number of patients, n** | | 1816 | | 1444 (80 %) | | 372 (20 %) | |
| **Median (IQR) SJC46** | 1723 (95 %) | 6 (3, 10) | 1371 (95 %) | 5 (2, 9) | 352 (95 %) | 10 (6, 15) | <0.001 |
| **Median (IQR) TJC46** | 1723 (95 %) | 6 (3, 11) | 1371 (95 %) | 5 (2, 10) | 352 (95 %) | 10 (5, 18) | <0.001 |
| **Proportions of patients with different SJC46 n, %** | | | | | | | |
| ≤2 | | 394 (23 %) | | 362 (26 %) | | 32 (9 %) | <0.001 |
| 3-5 | | 434 (25 %) | | 339 (28 %) | | 55 (16 %) | |
| ≥6 | | 894 (52 %) | | 629 (46 %) | | 265 (75 %) | |
| **Proportions of patients with different TJC46 n, %** | | | | | | | |
| ≤2 | | 404 (23 %) | | 356 (26 %) | | 46 (14 %) | 0.014 |
| 3-5 | | 435 (25 %) | | 378 (28 %) | | 57 (16 %) | |
| ≥6 | | 884 (51 %) | | 637 (46 %) | | 247(70 %) | |
| **Proportions of patients with symmetrical swelling by anatomical site n, %** | | | | | | | |

| | | | | | | | |
|---|---|---|---|---|---|---|---|
| Any site | 1624 (89 %) | 1214 (75 %) | 1285 (89 %) | 906 (71 %) | 339 (91 %) | 308 (91 %) | <0.001 |
| PIPs | | 537 (33 %) | | 375 (29 %) | | 162 (48 %) | <0.001 |
| MCPs | | 568 (35 %) | | 377 (29 %) | | 191 (56 %) | <0.001 |
| Wrists | | 550 (34 %) | | 365 (28 %) | | 190 (56 %) | <0.001 |
| Elbows | | 60 (4 %) | | 34 (3 %) | | 26 (8 %) | <0.001 |
| Knees | | 193 (12 %) | | 124 (10 %) | | 69 (20 %) | <0.001 |
| Ankles | | 136 (8 %) | | 73 (6 %) | | 63 (19 %) | <0.001 |
| MTPs | | 567 (35 %) | | 435 (34 %) | | 132 (39 %) | 0.097 |
| **Median (IQR) CRP** | 1717 (95 %) | 8 (3, 25) | 1361 (94 %) | 7 (3, 20) | 356 (96 %) | 19 (5, 46) | <0.001 |
| **Median (IQR) ESR** | 1536 (85 %) | 22 (10, 39) | 1221 (85 %) | 20 (10, 37) | 315 (85 %) | 26 (12, 46) | 0.006 |
| **Proportions of patients with normal CRP or ESR n, %** | | | | | | | |
| CRP | | 903 (53 %) | | 777 (57 %) | | 126 (35 %) | <0.001 |
| ESR | | 929 (60 %) | | 768 (63 %) | | 191 (51 %) | <0.001 |
| **Median (IQR) DAS28** | 1644 (91 %) | 3.9 (3.1, 4.7) | 1308 (91 %) | 3.7 (3.0, 4.5) | 336 (90 %) | 4.7 (4.0, 5.7) | <0.001 |
| **Mean (SD) DAS28** | | 4.0 (1.2) | | 3.7 (1.1) | | 4.8 (1.3) | <0.001 |
| **Median (IQR) Dr.global** | 1543 (85 %) | 40 (26, 56) | 1233 (85 %) | 39 (25, 50) | 310 (83 %) | 50 (38, 69) | <0.001 |
| **Median (IQR) PROs** | | | | | | | |
| Pain | 1593 (88 %) | 54 (30, 75) | 1272 (88 %) | 51 (30, 74) | 321 (86 %) | 61 (39, 77) | <0.001 |
| Fatigue | 1418 (78 %) | 45 (18, 70) | 1128 (78 %) | 40 (14, 69) | 290 (78 %) | 52 (31, 73) | <0.001 |
| PGA | 1590 (88 %) | 50 (25, 67) | 1273 (88 %) | 48 (23, 64) | 317 (85 %) | 54 (32, 70) | <0.001 |
| **Median (IQR) HAQ** | 1432 (79 %) | 0.90 (0.50, 1.40) | 1144 (79 %) | 0.88 (0.38, 1.4) | 288 (77 %) | 1.10 (0.75, 1.50) | <0.001 |

| | | | | | | | |
|---|---|---|---|---|---|---|---|
| **Mean (SD) HAQ** | | 0.97 (0.69) | | 0.93 (0.69) | | 1.20 (0.66) | |
| **Initial Medication** | | | | | | | |
| **MTX monotherapy** | | | | 242 (17 %) | | 86 (23 %) | |
| **MTX and csDMARD** | | | | 1039 (72 %) | | 249 (67 %) | |
| **only csDMARD** | | | | 159 (11 %) | | 36 (10 %) | |
| **bDMARD** | | | | 3 (0 %) | | 1 (0 %) | |
| **JAK-inhibitor** | | | | 1 (0 %) | | | |
| **GC** | | | | 1181 (82 %) | | 348 (94 %) | |

**Table 2. Clinical data of patients with RA at the initial presentation, by serological status**

## Effect of duration of symptoms on the clinical picture

Between patient groups by duration of symptoms, there were notable differences in terms of ACPA-positivity, DAS28, ESR, pain and HAQ. For other variables, such as SJC46, TJC46, CRP, Dr.global, fatigue and PGA, the differences between groups were minor and mostly statistically not significant (S2 Table).

*ACPA-positivity* was the most prevalent with a proportion of 77 % in group 3, compared to 70 % and 70 % % in other groups (p=0.045, adjusted for age and sex). There were similar results also for seropositivity (78 %, 77 % and 82 % in groups 1,2 and 3, p=0.115)- and RF-positivity (68 %, 67 % and 71 % in groups 1, 2 and 3, p=0.324) (S2 Table).

Commented [TS2]: sero what

*Disease activity.* The median (IQR) DAS28 was 4.1 (3.3, 5.1) in group 1, 4.0 (3.2, 4.8) in group 2 and 3.8 (3.1, 4.5) in group 3 (p=0.016, adjusted for age and sex). The median (IQR) ESR was 27 (12, 44) in group 1, 20 (10, 36) in group 2 and 18 (9, 34) in group 3 (p<0.001, adjusted for age and sex) and the proportions of patients with a normal level of ESR were 55 %, 61 % and 66 % in the same groups, respectively (p=0.046, adjusted for age and sex) (S2 Table).

*PROs.* For median (IQR) pain, the values in groups 1, 2 and 3 were 62 (35, 78), 52 (30, 72) and 50 (26, 70) (p<0.001, adjusted for age and sex). For median (IQR) HAQ, the values in the corresponding groups were 1.10 (0.50, 1.60), 0.9 (0.50, 1.40) and 0.75 (0.38, 1.20) (p=0.002, adjusted for age and sex) (S2 Table).

## Clinical variables between ACPA-positive but RF-negative and RF-positive but ACPA-negative patients

A total of 163 (11 % of seropositive patients) were only ACPA-positive and 118 (8 % of seropositive patients) were only RF-positive. The mean (SD) age was 53 (17) for solely ACPA- and 62 (14) for solely RF-positive patients (p<0.001) and the duration of symptoms for the same groups were 6 (3, 13) and 4 (2, 7) (p=0.222). The median (IQR) DAS28 was 3.6 (2.8, 4.2) for only ACPA-positive patients and 4.2 (3.5, 5.0) for only RF-positive patients (p<0.001). The same numbers for median (IQR) HAQ were 0.62 (0.3, 1.1) and 1.1 (0.5, 1.5) (p=0.033). Besides DAS28 and HAQ, there were no statistically significant differences, though all of the clinical variables were higher for only RF-positive patients (Table 3).

Commented [TS3]: TARKISTA TAULUJEN NUMEROT – KOMMENTEISSA KIRJOITAT ESIM S2, TÄMÄ TAULU ON 3 – ON VÄHÄN EPÄJOHDONMUKAISUUTTA

| Variable | Available data for ACPA-positive patients | ACPA-positive | Available data for RF-positive patients | RF-positive | p-value |
|---|---|---|---|---|---|
| **n, % of seropositive patients** | | 163 (11 %) | | 118 (8 %) | |
| **Age** | | 53 (17) | | 62 (14) | <0.001 |
| **Sex** | | 112 (69 %) | | 84 (71 %) | |
| **Duration of symptoms** | 124 (76 %) | 6 (3, 13) | 87 (74 %) | 4 (2, 7) | 0.222 |

| | | | | | |
|---|---|---|---|---|---|
| **Median (IQR) SJC46** | 156 (96 %) | 4 (2, 8) | 113 (96 %) | 6 (3, 10) | 0.193 |
| **Median (IQR) TJC46** | 156 (96 %) | 4 (2, 7) | 113 (96 %) | 7 (3, 12) | 0.056 |
| **Median (IQR) CRP** | 152 (93 %) | 5 (3, 13) | 111 (94 %) | 9 (3, 26) | 0.458 |
| **Median (IQR) ESR** | 137 (84 %) | 13 (7, 28) | 99 (84 %) | 20 (10, 35) | 0.780 |
| **Median (IQR) DAS28** | 148 (91 %) | 3.6 (2.8, 4.2) | 108 (92 %) | 4.2 (3.5, 5.0) | <0.001 |
| **Median (IQR) Dr.global** | 137 (84 %) | 35 (25, 50) | 103 (87 %) | 40 (27, 50) | 0.194 |
| **Median (IQR) PROs**<br>**Pain**<br>**Fatigue**<br>**PGA** | 146 (90 %)<br>131 (80 %)<br>146 (90 %) | 50 (27, 75)<br>50 (20, 70)<br>50 (25, 64) | 104 (88 %)<br>91 (77 %)<br>103 (87 %) | 58 (33, 77)<br>46 (18, 74)<br>51 (32, 70) | 0.145<br>0.767<br>0.402 |
| **Median (IQR) HAQ** | 134 (82 %) | 0.62 (0.3, 1.1) | 93 (79 %) | 1.1 (0.5, 1.5) | 0.033 |

**Table 3. Clinical data of patients that were only ACPA- or RF-positive.**

# Discussion

Our main observation was that early RA is still mainly presented as symmetric arthritis, as three out of four patients had symmetrical swelling in any of the anatomical sites of the 1987 criteria at the initial presentation (1). Overall, the wrists were the most commonly swollen joints by a significant margin (S1 Table). Symmetrical swelling was detected more often overall and at all sites in ACPA negative patients. This was an expected finding since more swollen joints are required to fulfill the criteria for seronegative RA (5). Seronegative patients had also higher SJC46 at the initial presentation (median 5 *versus* 10). Interestingly, the presence of symmetrical swelling was only slightly higher for seronegative patients in the MTPs (34 % *versus* 39 %, p=0.097) (Table 2) despite higher SJC46.

*Clinical measures and medication.* In terms of clinical data, seronegative RA presented significantly higher activity by almost all variables used in the study (Table 2). Similar differences were found between solely ACPA-positive and solely RF-positive patients, though most of the findings weren't statistically significant (Table 3). In ESR, the difference was only minor between seropositive and seronegative patients (20 *versus* 24, p=0.172) (Table 2). Seropositive patients were more likely to fulfill the ACR/EULAR 2010 criteria (82 % *versus* 78) at the time of diagnosis. Patients with seropositive RA were also current smokers more often, (21 % *versus* 12 % current smokers) (Table 1), as smoking is a known risk-factor for ACPA-positive RA (24). In terms of medication, GCs and MTX as monotherapy was used more frequently in patients that were diagnosed with seronegative RA. The more prevalent use of GCs indicates that some of the diagnoses might have been actually polymyalgia rheumaticas, as our previous studies have suggested (10,11).

**Commented [LW4]:** Viitteitä johonkin vai pelkka pohdinta???

*Comparison to other studies.* The results between seropositive and negative RA were somewhat contradictory compared to earlier research, a change likely caused by aiming at earlier diagnosis in accordance with the 2010 classification criteria. A French study of 354 patients diagnosed between 2002 and 2005 showed that seropositive patients had higher average DAS28 (5.3 *versus* 5.0) and HAQ (1.0 *versus* 0.9) than seronegative patients, but similar joint counts and VAS-values of pain (12). In our study, the variables were significantly higher for seronegative RA (mean DAS28 of 4.8 *versus* 3.7 and 1.2 *versus* 0.93 for HAQ). However, the populations are not directly comparable, as the study had slightly different threshold values for ACPA and RF.

A Danish study of 198 newly diagnosed seropositive and 36 seronegative patients diagnosed in 2010 to 2013 showed lower median SJC44 (8 *versus* 17), Dr.global (39 *versus* 49) and DAS28 (3.4 *versus* 3.9), but similar PROs at the initial presentation in ACPA positive *versus* negative patients (25). On the contrary, our population showed significantly lower PROs at the initial presentation in seropositive *versus* seronegative patients (51 *versus* 61 for pain, 40 *versus* 52 for fatigue and 48 *versus* 54 for PGA) (Table 2), in addition to other clinical variables.

In terms of duration of symptoms, a previous study of patients diagnosed in 2007-2012 in Canada showed a duration of symptoms of 6.5 months for patients with seropositive RA and 5.4 months for patients with seronegative RA (26). This is in line with our results, where the median duration of symptoms was five months for seropositive and four months for seronegative RA (Table 1).

*The effect of duration of symptoms on the initial presentation.* The overall median SJC46 was the same in all groups by duration of symptoms (median 6 joints) (S2 Table) and median DAS28 was higher in the group of patients with a delay of < 3 months compared to patients with a delay of >6 months (4.1 *versus* 3.8, p=0.016, adjusted for age and sex). Interestingly, patients with a delay of <3 and 3-6 months were also older than in the group with a delay of >6 months (mean age of 61 and 58 in groups with a delay of <3 and 3-6 months and 56 in the group with a delay of >6 months, p<0.001) (S2 Table). It has been shown that older patients utilize health care services more frequently, which might explain the finding (27). The overall median CRP and ESR were higher in patients who were diagnosed earlier (11 in the group with a delay of <3 months, *versus* 8 and 7 in groups with a delay of 3-6 months and >6 months for CRP) and (27 *versus* 20 and 18 for ESR in the same groups, correspondingly). High laboratory values might have influenced towards early referrals, which in turn lowers the duration of symptoms. In addition, the median VAS-pain and HAQ were also significantly higher in patients who were diagnosed earlier (62 in the group with a delay of <3 months *versus* 52 and 50 in groups with 3-6 and >6 months, for pain) and (1.10 *versus* 0.90 and 0.75 for the same groups for HAQ), but ACPA was positive less frequently (77 % in the group with a delay of >6 months *versus* 70 % and 70 % in other groups, p=0.045) (S2 Table), which indicates that patients experiencing more severe symptoms and limitations in physical activity might seek health care services earlier. ACPA-positivity isn't necessarily associated with the severity of symptoms. A previous Polish study has shown that the second most important factor for the duration of symptoms of RA is patients' conviction that the condition will resolve on its own (28).

## Strengths and limitations

*The main strength* of this study was its large patient population from almost all health care regions, in Finland and that patient data was documented recently allowing an accurate presentation of the current clinical picture.

*Weaknesses.* Although all health care regions are included in the quality register, at the time that was defined as the focus period, not all regions actively recorded clinical data. Therefore, probably not all patients with incident RA could be included. Furthermore, although not recommended, patients may still be diagnosed along with receiving treatment initiation in private practice, which are not involved in the quality register, yet.

One of the weaknesses in register studies is missing data, although the completeness was as high as ≥95% for joint counts and ≥78 % for PROs.

## Conclusions

Our results indicate that the initial presentation of early RA is still mostly symmetric seropositive arthritis. Furthermore, we found that seronegative RA has significantly higher inflammatory activity and disease burden at the initial presentation by several clinical variables, compared to seropositive cases. However, 25 % of patients in the current study didn't have symmetrical swelling at the initial presentation, which is why all patients with clinical joint inflammation of unknown reason ought to be tested for ACPA and RF and referred to a rheumatology unit if ACPA and/or RF ispositive. Our observations are encouraging in terms that patients experiencing more severe pain and decrease in functional ability might seek health care services earlier. We also found that ACPA--positivity is not necessarily associated with the severity of symptoms.

## References:

1. Arnett F. Classification criteria: The 1987 American Rheumatism Association revised criteria for the classification of rheumatoid arthritis. CPD Rheumatol. 1999;1(1):13–4.

2. Bukhari M, Lunt M, Harrison BJ, Scott DGI, Symmons DPM, Silman AJ. Erosions in inflammatory polyarthritis are symmetrical regardless of rheumatoid factor status: Results from a primary care-based inception cohort of patients. Rheumatology. 2002;41(3):246–52.

3. Zangger P, Keystone EC, Bogoch ER. Asymmetry of small joint involvement in rheumatoid arthritis: Prevalence and tendency towards symmetry over time. Jt Bone Spine. 2005;72(3):241–7.

4. Sanchez-Castillo JA, Reinoso-Cobo A, Gijon-Nogueron G, Caliz-Caliz R, Exposito-Ruiz M, Ramos-Petersen L, et al. Symmetry criterion for patients with rheumatoid arthritis of the foot: A cross-sectional study. Int J Environ Res Public Health. 2021;18(7).

5. Aletaha D, Neogi T, Silman AJ, Funovits J, Felson DT, Bingham CO, et al. 2010 Rheumatoid arthritis classification criteria: An American College of Rheumatology/European League Against Rheumatism collaborative initiative. Arthritis Rheum. 2010;62(9):2569–81.

6. Van Der Helm-Van Mil AHM, Zink A. What is rheumatoid arthritis? Considering consequences of changed classification criteria. Ann Rheum Dis. 2017;76(2):315–7.

7. Raychaudhuri S. NIH Public Access. Curr Opin Rheumatol. 2010;22(2):109–18.

8. Chang K, Yang SM, Kim SH, Han KH, Park SJ, Shin J Il. Smoking and rheumatoid arthritis. Int J Mol Sci. 2014;15(12):22279–95.

9. Asikainen J, Nikiphorou E, Kaarela K, Lindqvist E, Häkkinen A, Kautiainen H, et al. Is long-term radiographic joint damage different between men and women? Prospective longitudinal data analysis of four early RA cohorts with greater than 15 years of follow-up. Vol. 34, Clinical and Experimental Rheumatology. 2016.

10. Paalanen K, Rannio K, Rannio T, Asikainen J, Hannonen P, Sokka T. Does early seronegative arthritis develop into rheumatoid arthritis? A 10-year observational study. Clin Exp

Rheumatol. 2019;37(1):37–43.

11. Paalanen K, Puolakka K, Nikiphorou E, Hannonen P, Sokka T. Is seronegative rheumatoid arthritis true rheumatoid arthritis? A nationwide cohort study. Rheumatol (United Kingdom). 2021;60(5):2391–5.

12. Mouterde G, Rincheval N, Lukas C, Daien C, Saraux A, Dieudé P, et al. Outcome of patients with early arthritis without rheumatoid factor and ACPA and predictors of rheumatoid arthritis in the ESPOIR cohort. Arthritis Res Ther. 2019;21(1):1–9.

13. Luukkainen R, Kajander A, Isomaki H. Treatment of rheumatoid arthritis. Vol. 2, British Medical Journal. 1978. p. 1501.

14. Emery P, Salmon M. Early rheumatoid arthritis: Time to aim for remission? Ann Rheum Dis. 1995;54(12):944–7.

15. Smolen JS, Aletaha D, Bijlsma JWJ, Breedveld FC, Boumpas D, Burmester G, et al. Treating rheumatoid arthritis to target: Recommendations of an international task force. Ann Rheum Dis. 2010;69(4):631–7.

16. Gremese E, Salaffi F, Bosello SL, Ciapetti A, Bobbio-Pallavicini F, Caporali R, et al. Very early rheumatoid arthritis as a predictor of remission: A multicentre real life prospective study. Ann Rheum Dis. 2013;72(6):858–62.

17. Quinn MA, Emery P. Window of opportunity in early rheumatoid arthritis: Possibility of altering the disease process with early intervention. Clin Exp Rheumatol. 2003;21(5 SUPPL. 31).

18. Sørensen J, Hetland ML. Diagnostic delay in patients with rheumatoid arthritis, psoriatic arthritis and ankylosing spondylitis: Results from the Danish nationwide DANBIO registry. Ann Rheum Dis. 2015;74(3):1–7.

19. Van Der Linden MPM, Le Cessie S, Raza K, Van Der Woude D, Knevel R, Huizinga TWJ, et al. Long-term impact of delay in assessment of patients with early arthritis. Arthritis Rheum. 2010;62(12):3537–46.

20. Mankia K, Nam J, Emery P. Identifying arthralgia suspicious for progression to rheumatoid arthritis. Ann Rheum Dis. 2017;76(6):e14.

21. Prevoo MLL, Van'T Hof MA, Kuper HH, Van Leeuwen MA, Van De Putte LBA, Van Riel PLCM. Modified disease activity scores that include twenty-eight-joint counts development and validation in a prospective longitudinal study of patients with rheumatoid arthritis. Arthritis Rheum. 1995;38(1):44–8.

22. Krishnan E, Sokka T, Häkkinen A, Hubert H, Hannonen P. Normative Values for the Health Assessment Questionnaire Disability Index: Benchmarking Disability in the General Population. Arthritis Rheum. 2004;50(3):953–60.

23. Sokka T, Toloza S, Cutolo M, Kautiainen H, Makinen H, Gogus F, et al. Women, men, and rheumatoid arthritis: Analyses of disease activity, disease characteristics, and treatments in the QUEST-RA Study. Arthritis Res Ther. 2009;11(1).

24. Hedström AK, Stawiarz L, Klareskog L, Alfredsson L. Smoking and susceptibility to rheumatoid arthritis in a Swedish population-based case–control study. Eur J Epidemiol. 2018;33(4):415–23.

25. Nordberg LB, Lillegraven S, Lie E, Aga AB, Olsen IC, Hammer HB, et al. Patients with seronegative RA have more inflammatory activity compared with patients with seropositive RA in an inception cohort of DMARD-naïve patients classified according to the 2010 ACR/EULAR criteria. Ann Rheum Dis. 2017;76(2):341–5.

26. Barra L, Pope JE, Orav JE, Boire G, Haraoui B, Hitchon C, et al. Prognosis of seronegative patients in a large prospective cohort of patients with early inflammatory arthritis. J Rheumatol. 2014;41(12):2361–9.

27. Nie JX, Wang L, Tracy CS, Moineddin R, Upshur RE. Health care service utilization among the elderly: Findings from the Study to Understand the Chronic Condition Experience of the Elderly and the Disabled (SUCCEED project). J Eval Clin Pract. 2008;14(6):1044–9.

28. Raciborski F, Kłak A, Kwiatkowska B, Batko B, Sochocka-Bykowska M, Zoń-Giebel A, et al. Diagnostic delays in rheumatic diseases with associated arthritis. Reumatologia. 2017;55(4):169–76.

Click here to access/download
**Supporting Information**
Revised_table.docx

Click here to access/download
**Supporting Information**
Revised_table_with_track_changes.docx

Central Finland Health Care District, UEF

# Initial presentation of early rheumatoid arthritis

Formatted: Font: 18 pt

Lauri Weman[1]*, Henri Salo [2], Laura Kuusalo [3], Johanna Huhtakangas [4], Johanna Kärki [5], Paula Vähäsalo [6], Maria Backström [7]

Tuulikki Sokka-Isler [8]

[1] Faculty of Health Sciences, University of Eastern Finland, Kuopio, Finland

[2] Finnish institute for health and welfare (THL), Helsinki, Finland

[3] Centre for Rheumatology and Clinical Immunology, Turku University Hospital, Turku, Finland

[4] Division of Rheumatology, Kuopio University Hospital, Kuopio, Finland

[5] Department of Children and Adolescents, Kanta-Häme Central Hospital, Hämeenlinna, Finland

[6] PEDEGO Research Unit, University of Oulu, Oulu, Finland

[7] Department of Pediatrics, Vaasa Central Hospital, Vaasa, Finland

[8] Department of Rheumatology, Central Finland Central Hospital, Jyväskylä, Finland

*Corresponding author

E-mail:lauriweman@gmail.com

This study received no external funding. None of the authors had any conflicts of interest.

MD Lauri Weman

M.Sc Henri Salo

Johanna Huhtakangas MD PHD, Laura Kuusalo, MD, PhD, Paula Vähäsalo, MD, PhD, Maria Backström, MD, PhD, Johanna Kärki, MD

Professor Tuulikki Sokka-Isler

Central Finland Central Hospital Rheumatology Outpatient Clinic

Hoitajantie 3 40620 Jyväskylä

# Abstract

Formatted: Font: 18 pt


*Objectives:* To study the joint distribution and clinical picture of rheumatoid arthritis (RA) at the initial presentation in~~ anti-citrullinated protein antibody (ACPA) ~~seropositive (anti-citrullinated protein antibody (ACPA) and/or rheumatoid factor (RF) positive) and negative patients and the effect of duration of symptoms~~iagnostic delay~~ on the clinical picture.

*Methods:* Data of patients who received reimbursement for DMARDs for newly diagnosed RA in 1/2019 to 9/2021 were extracted from the national databases. ~~Swollen and tender joint~~Joint counts, presence of symmetrical swelling, other disease activity measures, and patient reported outcomes (PROs) were compared in sero~~ACPA ~~positive and negative patients. Regression analyses were applied to compare clinical variables in patients with duration of symptoms of <3, 3-6, and >6 months, adjusted for age, sex, and ~~ACPA positivity~~seropositivity.

*Results:* Data of 1816~~01~~ ACPA and RF~~-~~-tested patients were included. Symmetrical swelling was present in 75 % of patients. Sero~~ACPA ~~negative *versus* positive patients had higher value for all disease activity measures and PROs including median swollen joint count (SJC46 10~~9~~ *versus* 5) and DAS28 (4.7~~5~~ *versus* 3.7), (p<0.001). Patients diagnosed in <3 months had higher median pain VAS (62 *versus* 52 and 50, p<0.001) and HAQ (1.1 versus 0.9 and 0.75, p=0.002~~3~~) compared to those with a duration of symptoms of 3-6 and >6 months. Patients diagnosed >6 months were ~~ACPA ~~ACPA-~~sero~~positive more frequently (77~~6~~ % *versus* 70~~69~~ % in other groups, p=~~<~~0.045~~01~~).

*Conclusion:* Incident RA presents mainly as symmetric arthritis. Sero~~ACPA ~~negative patients have higher disease burden at the initial presentation. Patients experiencing more severe pain and decreased functional ability are diagnosed earlier, regardless of ~~serological~~ACPA- ~~ACPA ~~status.

# Background

Formatted: Font: 18 pt

Before the introduction of modern diagnostic tools, incident rheumatoid arthritis (RA) was recognized as symmetric polyarthritis.  Symmetry was one criterion in the previous 1987 classification criteria for RA (1), which were developed to differentiate RA from other types of inflammatory arthritides.  Symmetry of joint erosions has been observed in patients with early RA diagnosed in the early 1990's (2) and in patients with established RA in 1963-1996 (2,3). However, no studies have been conducted recently on the symmetry of joint involvement at the presentation of RA (4). Although not part of the current classification criteria (5), symmetry of arthritis is still being taught in textbooks and medical schools, as one of the hallmarks of incident RA. However, considering RA only in cases with symmetric polyarthritis might postpone the suspicion of early RA in primary health care.

In the 2010 ACR/EULAR classification criteria for RA, more emphasis has been placed on serology (5) without requirement for symmetry. With the 2010 criteria, early RA is now presented mainly in two forms with 6 criteria fulfilled, either seropositive RA, which can be oligoarthritis without elevated inflammatory markers, or seronegative RA, which is usually polyarthritis with elevated inflammatory markers. In fact, it is getting obvious that autoantibodies are the hallmark of RA and seronegative RA is a distinct entity (6) with different risk factors (7,8) and outcomes compared to seropositive RA (9). Our recent study indicated that seronegative RA turns out to be a spectrum of different conditions when observed for 10 years (10,11). Nonetheless, seronegative active arthritis requires early identification and tightly controlled treatment with Disease Modifying Antirheumatic Drugs (DMARDs) as it also causes significant treatable disease burden to patients (12).

First  in the 1970's (13) and more widely since the 1990's, it has been recognized that RA should be identified and its treatment  initiated as soon as possible to facilitate good outcomes (13,14). With multiple studies showing that early recognition is crucial, it has since been emphasized as an important approach for the management of RA (15). It has also been shown that treatment initiation within the first 12 weeks after the first symptoms might be the best window of opportunity in terms of treatment outcomes (16,17). Some studies suggest that the duration of symptoms~~iagnostic delay~~ has decreased during the current millennium (18). However, there are still notable delays in terms of treatment initiation. For example, only 31 % of patients with incident RA were examined by a

rheumatologist within 12 weeks of the first symptoms in the Netherlands in 1993-2006 (19). In the UK, patients with early RA visit their general practitioner 4 times on average before being referred to a specialist  according to a report from 2008-2009 (20).

Therefore, our objective was to study the initial presentation of RA in a nationwide setting, with a focus on the ~~ACPA~~ serological status and symmetry of joint swelling, as well as the effect of ~~d~~duration of symptoms~~iagnostic delay~~.

# Methods

Formatted: Font: 18 pt

**Setting.** Patients with incident RA are diagnosed and treated in rheumatology outpatient clinics in Finland. According to the national guidelines, DMARDs are started at the time of the diagnosis together with a medication reimbursement application, prepared by the rheumatologist. The reimbursement is granted by the Social Insurance Institution of Finland (KELA) for DMARDs. In addition to granting reimbursements for patients with chronic illnesses, such as RA, KELA also maintains a database of the individuals, containing the ICD code of the diagnosis, date of the reimbursement and basic demographic data of the individuals.

Formatted: Font: 16 pt

For the current study, patients who received their first reimbursement for DMARDs prescribed for the treatment of RA between 1/2019-9/2021 were identified in the KELA database. Clinical and demographic data were extracted from The Finnish Rheumatology Quality Register, using the individual identification code. To capture the data at the time of the diagnosis, clinical data were extracted from the most recent visit within 0 to 90 days prior to the date of the medication reimbursement; visits occurred between 23rd November 2018 and 30th August 2021. All patients were over 16 years old and could not be diagnosed as any other specific arthritis. The ACR/EULAR 2010 classification criteria were used to aid in diagnosing the patients (5).

~~**Cases.** The focus of this study is ACPA status, instead of seropositivity, which also includes rheumatoid factor (RF) positive, but ACPA-negative patients. The reason for this was that RF has been shown to be elevated in many other connective tissue disorders, bacterial and viral infections, malignancies and liver diseases (22), it is a less specific marker for RA (23) and ACPA-positivity is a stronger predictor of a more severe disease course and erosive disease (24).~~

**Variables.** Following variables were collected at the time of the diagnosis of RA and were available for analyses.

Formatted: Font: 16 pt

*Demographic variables*

- Age in years
- Sex; male subjects/female subjects
- Smoking status: current smoking; having ever smoked
- Employment status for patients under 65 years old; currently working, unemployed, disabled and not in work force such as home makers and students

*Disease characteristics*

- Duration of symptoms
- Fulfillment of ACR/EULAR 2010-criteria (5).

*Clinical variables*

- Swollen joint count (SJC) and Tender joint count (TJC) on 46 joint counts
- Distribution of swollen and tender joints on 46 joint counts
- Presence of symmetrical swelling in the MCPs, PIPs, wrists, MTPs, knees, ankles, and the elbows
- C-reactive protein (CRP), erythrocyte sedimentation rate (ESR) and DAS28 scores
- Investigator's global assessment of disease activity (Dr.global)
- Patient reported outcomes (PROs) such as pain, fatigue, and Patient Global Assessment (PGA)
- Self-reported functional capacity according to the Stanford Health Assessment Questionnaire (HAQ).

Formatted: Font: 12 pt

*The Initial Medication*

Formatted: Font: Italic

- The medications of interest were ~~prescription of~~ methotrexate (MTX), other conventional synthetic disease-modifying antirheumatic drugs (csDMARDs), such as hydroxychloroquine

Formatted: List Paragraph, Bulleted + Level: 1 + Aligned at: 0.25" + Indent at: 0.5"

(HCQ) and sulfasalazine (SSZ), prednisolone (GC), biological DMARDs (bDMARDs) and janus kinase (JAK) inhibitors, as well as systemic glucocorticoids (GC).

Formatted: Font: 12 pt

*Duration of symptoms*, which is the time between the first symptoms and the diagnosis of RA , recorded in months. At the initial visit, the patient was asked when he/she recognized the first symptoms of RA. from the patient and it was recorded in months.

*ACPA- positivity* was defined by the laboratory reference values, which was ≥ 7 kU/l. In this study, RF-positivity wasn't included in the analyses.

*RF-positivity* was defined by the laboratory reference values, being ≥ 15 IU/ml.

*Fulfilment of the ACR/EULAR 2010 criteria* was calculated by the examining physician, who counts the items in the ACR/EULAR 2010-criteria. If the total score adds up to 6 or more points, the ACR/EULAR 2010-criteria are met, when other reasons that can explain the condition are excluded(a score of ≥6/10) was calculated (5).

*Joint counts.* The presence of swollen and tender joints was calculated using a 46 joint count including proximal interphalangeal (PIP), and metacarpophalangeal (MCP) joints, interphalangeal (IP) joints of the thumbs, wrists, metatarsophalangeal (MTP) joints, distal interphalangeal (DIP) joints of big toes, temporomandibular joints as well as ankle-, knee-, hip-, elbow- and shoulder joints.

*Symmetrical swelling* was analyzed from the MCPs, PIPs, wrists, MTPs, knees, ankles and the elbows, according to the 1987 criteria (1). A patient was considered to have symmetrical swelling if he/she had bilateral swelling in the same anatomical site. Symmetrical joint tenderness wasn't analyzed as it wasn't in the 1987 criteria (1).

*CRP and ESR* were determined according to the laboratory reference values for women and men in different age groups. The A reference value of below 10 mg/l was normal for CRP. An elevated ESR included values of ≥ 20 mm/h for women younger than 50 years, ≥ 30 mm/h for women over 50 years and ≥42 mm/h for women over 85 years and ≥ 15 mm/h for men younger than 50 years, ≥20 mm/h for men over 50 years and ≥30 mm/h for men over 85 years.

*DAS28* was used to describe disease activity (21).

*Dr.global* included the physicians' assessment of rheumatic activity on the 0-100 mm Visual Analog Scale (VAS), where 0 equals no disease activity and 100 maximal disease activity.

*PROs* included the self-assessment of pain, fatigue, and PGA on the 0-100 mm VAS, where 0 equals no symptoms and 100 maximal discomfort.

*HAQ* was used to describe the functional capacity of patients. It is scored from 0 to 3, a score of <0.5 is a sign of good functional status. In this study, we used HAQ without ''aids and devices'' due to its better accuracy in clinical studies. (22,23)

*The Initial Medications* were analyzed in following groups:

**Formatted:** Font: Not Italic

- MTX monotherapy
- MTX in combination with (an)other csDMARD(s)
- csDMARD monotherapy or a combination
- bDMARDbDMARD with or without MTX
- JAK- inhibitor with or without MTX
- GC, regardless of DMARDs

**Formatted:** List Paragraph, Bulleted + Level: 1 + Aligned at: 0.25" + Indent at: 0.5"

**Formatted:** Font: Not Italic

. The use of medication was analyzed according to what type of disease-modifying antirheumatic drug drug(DMARD), or multiple drugsdrugs were prescribed for the patient for the treatment of RA at their initial visit. The focus was on the use of methotrexate (MTX) as monotherapy, MTX and any other conventional synthetic DMARD (csDMARD), such as sulfasalazine or hydroxychloroquine, use of other csDMARDs than MTX, any kind of prednisolone (GC) usage and the use of biological DMARDs (bDMARDs) or janus kinase inhibitor (JAK-inhibitors) as the initial medication.

**Commented [TS1]:** EI VOI KÄYTTÄÄ PRESCRIBED KOSKA EIHÄN ME NIITÄ TIEDETÄ, TIEDETÄÄN VAIN OSTOT.

Clinical and demographic variables were determined for all patients and for seropositive and negative patients. Comparisons were conducted between seropositive and negative patients as well as between only ACPA-positive and only RF-positive patients with clinical variables. The use of medication was determined only for seropositive and seronegative patients and their use was compared between these groups.

**Methods to study the effect of duration of symptomsiagnostic delay on disease activity.** Three groups were formed according to the duration of symptoms before receiving the diagnosis: patients with a duration of symptomsiagnostic delay of <3 months (group 1), patients with a duration of symptomsdelay of 3-6 months (group 2) and patients with a duration of symptomsiagnostic delay of over 6 months (group 3). SJC46, TJC46, ACPA serological status, CRP, ESR, DAS28, PROs, HAQ, and DDr.global were compared between these groups.

Formatted: Font: 16 pt

**Statistical methods.** Descriptive statistics were used with mean values with standard deviation (SD) and median values with interquartile ranges (IQR) depending on the way a value is distributed. Chi-square test was used to compare categorical variables. P=0.05 was set as a threshold for statistical significance. Regression models were used to compare clinical and demographic variables, ANOVA for continuous variables and logistic regression for dichotomous correlatives and for comparisons of median values transformed as median splits. Adjustments for age and sex were used in the comparisons of patients by ACPA-status. Variables were adjusted for ACPAsero-positivity, age and sex in the comparisons of groups by duration of symptomsiagnostic delay.

Formatted: Font: 16 pt

Analyses were conducted using the R Statistical language (version 4.2.1; R Core Team, 2022) on Ubuntu 20.04.5 LTS.

## Ethical issues.

Formatted: Font: 16 pt

This study was conducted as a register-based study using data from the Finnish Rheumatology Quality Register, which is kept by the Finnish Institute for Health and Welfare (THL) and, which granted the approval for the study. In a register-based study, patient consent was not required.

# Results

Formatted: Font: 18 pt

## Demographic variables

Formatted: Font: 16 pt

A total of 2017 patients with incident RA were identified in the database. ACPA ~~and~~ and/or RF ~~whereas~~ were available for 181601~~01~~ (~~89~~ 90 %) patients, which were included in the analyses. Of these, 1444~~294~~ (80~~72~~ %) were sero~~ACPA~~-positive and 372~~507~~ (20~~8~~ %) were sero~~ACPA~~-negative. A total of 5 (0.3 %) seropositive patients weren't tested for ACPA, as they were already RF-positive. Vice-versa, 83 (6 %) patients weren't tested for RF, as they already had a positive ACPA-result. The mean (SD) age was 59 (16) years for all patients~~, 61 (14) for males and 58 (16) for females.~~ . For sero~~ACPA~~-positive and negative patients, the corresponding numbers were 59~~8~~ (15~~6~~) and 60 (16) (p<0.001)~~, 60 (14) and 57 (16). For ACPA negative patients, the same numbers were 61 (16), 63 (14) and 59 (16).~~ .A total of 1034~~28~~ (57 %) patients overall, 850 ~~77~~ ~~0~~ (59~~60~~ %) sero~~ACPA~~-positive patients and 184~~258~~ (49~~51~~ %) sero~~ACPA~~-negative patients were under 65 years old at the initial visit (p=0.002). (Table 1).

A total of 19 % of all patients were current smokers and 55 % had ever smoked. The corresponding proportions were 21 % and 57~~8~~ % for sero~~ACPA~~-positive patients and 12 % and 46 % for sero~~ACPA-negative~~ negative patients (p<0.001 for both comparisons, adjusted for age and sex). In terms of employment status, a total of 69 % of all patients were employed, 20 % disabled, 8~~7~~ % unemployed and 4 % weren't currently in work force. No major differences were found between sero~~ACPA~~ positive and negative patients. (Table 1).

## Disease characteristics

Formatted: Font: 16 pt

The median (IQR) duration of symptoms before being diagnosed was 4 (2, 10) for all patients. For sero~~ACPA~~-positive patients it was 5 (2~~3~~, 10) and 4 (2, 8) for sero~~ACPA~~-negative patients (p=0.03~~1~~0, adjusted for age and sex).

The proportions of patients fulfilling the ACR/EULAR 2010-criteria at the initial presentation were 88~~2~~ % for all patients, 91~~2~~ % for sero~~ACPA~~-positive and 74~~8~~ % for sero~~ACPA~~-negative patients (p<0.001, adjusted for age and sex) (Table 1).

| Variable | Available data for all patients | All | Available data for seroACPA positive n, (%) | SseroACPA positive | Available data for seroACPA negative n, (%) | SseroACPA negative | p-value |
|---|---|---|---|---|---|---|---|
| **n** | 181601 (100 %) | 181601 | 1294 (100 %) | 1444294 (8072 %) | 507 (100 %) | 372507 (2028 %) | |
| **Females, n (%)** | 181601 (100 %) | 11770 (65 %) | 1444294 (100 %) | 947845 (65 %) | 372507 (100 %) | 230325 (624 %) | |
| **Mean (SD) Age in years**<br>All<br>Men<br>Women | 181601 (100 %) | 59 (16)<br>61 (14)<br>58 (16) | 1444294 (100 %) | 58 (16)<br>60 (14)<br>57 (16) | 372507 (100 %) | 601 (16)<br>63 (14)<br>59 (16) | <0.001 |
| **Smoking status, n (%)**<br>Current smokers<br>Patients that have ever smoked | 1707690 (94 %) | 321315 (19 %)<br>942928 (55 %) | 1363226 (945 %) | 28160 (21 %)<br>78314 (578 %) | 344464 (92 %) | 5540 (12 %)<br>159214 (46 %) | <0.001<br><0.001 |
| **Patients under 65 years old, n (%)** | | 103428 (57 %) | | 850770 (5960 %) | | 184258 (4951 %) | <0.0021 |
| **Employment status for patients under 65 years old, n (%)**<br>Employed<br>Disabled<br>Unemployed<br>Not in work force | 73328 (71 %) | 5042 (69 %)<br>1486 (20 %)<br>5251 (87 %)<br>29 (4 %) | 601545 (71 %) | 415381 (6970 %)<br>11905 (2019 %)<br>4639 (87 %)<br>210 (34 %) | 132183 (7271 %) | 89121 (676 %)<br>2941 (22 %)<br>612 (57 %)<br>89 (65 %) | 0.273454 |
| **Disease characteristics** | | | | | | | |
| **Median (IQR) duration of symptomsdiagnostic delay in months** | 12887 (7194 %) | 4 (2, 10) | 1026928 (712 %) | 5 (23, 10) | 262359 (701 %) | 4 (2, 8) | 0.03010 |

Formatted: English (United States)

| | | | | | | | |
|---|---|---|---|---|---|---|---|
| **Proportions of patients fulfilling the ACR/EULAR 2010 criteria** | 1497 (823 %) | 1311 (88 %) | 1165058 (812 %) | 1064970 (912 %) | 332439 (897 %) | 247341 (748 %) | <0.001<0.001 |

**Table 1. Demographic data and disease characteristics of patients with RA at the initial presentation, by serological ACPA status.**

## Swollen- and tender joint counts

Formatted: Font: 16 pt

The median (IQR) SJC46 was 6 (3, 101) for all patients. For seroACPA positive patients it was 5 (2, 9) and 109 (65, 154) for seroACPA negative patients (p<0.001). The values for TJC46 were 6 (3, 11) for all patients and 5 (2, 109) and 109 (54, 187) for seroACPA positive and negative patients, respectively (p<0.001, adjusted for age and sex) (Table 2).

For all patients, 23 % had two or less swollen joints at the initial presentation, 25 % had 3-5 and 52 % had ≥6 swollen joints on the 46 swollen joint count. The corresponding proportions were 267 %, 28 % and 465 % for seroACPA positive patients and 912 %, 168 % and 7569 % for seroACPA negative patients (p<0.001, adjusted for age and sex) (Table 2).

A total of 23 % of all patients had two or less tender joints in the 46 tender joint count, 25 % had 3-5 and 52 % ≥6 tender joints. For seroACPA positive patients the proportions were 26 %, 28 % and 46 % and 146 %, 168 % and 7066 % for seroACPA negative patients (p<0.001, adjusted for age and sex) (Table 2).

## Pattern and symmetry of joint involvement at the initial presentation

Formatted: Font: 16 pt

At the initial presentation, wrists were the most commonly affected joints, with 43.56 % of patients having a swollen left wrist and 43.65 % a swollen right wrist. Any joint from the right MCP's was swollen in 9.56 to 34.9 % of patients and in 9.7 to 28.78 % of patients from the left MCP's. The corresponding proportions were 9.56 to 31.57 % for the right PIP's and 8.23 to 25.45 % for the left PIP's and 10.34 to 28.02 % for the right and 11.0 to 27.67 % for the left MTPs. Knees were the most commonly swollen large joints (20.86 % for right and 17.21 % for the left). The corresponding proportions were 9.6 % and 78.90 % for shoulders, 13.52 % and 123.80 % for ankles, 6.87% and 5.98 % for elbows and 1.9 % and 2.23 % for the hips (S1 Table 3).

At the initial presentation of RA, a total of 75 % of all patients had symmetrical swelling in any of the anatomical sites. In different anatomical sites, 35 % had symmetrical swelling in MCPs, 33 % in the PIPs, 34 % in wrists, 35 % in the MTPs, 12 % in the knees, 8 % in the ankles and 4 % in the elbows. A total of 710 % of seroACPA positive patients had any symmetrical swelling, and the corresponding proportion was 9187 % for seroACPA negative patients (<0.001, adjusted for age and sex). In terms different anatomical sites, seroACPA negative patients had more symmetrical swelling in all sites,

with the biggest differences in the wrists compared to seroACPA positive patients (28 % in seroACPA positive and 561 % in seroACPA negative patients) (p<0.001, adjusted for age and sex) and the MCPs (29 % and 560 %) (p<0.001, adjusted for age and sex). A total of 34 % of seroACPA positive patients had symmetrical swelling in the MTPs and 396 % of seroACPA negative patients (p=0.097.547) (Table 2, Figure 1).

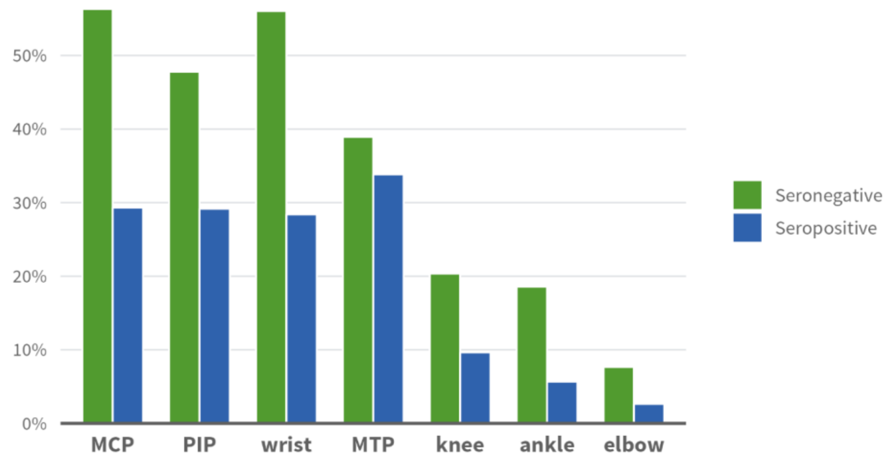


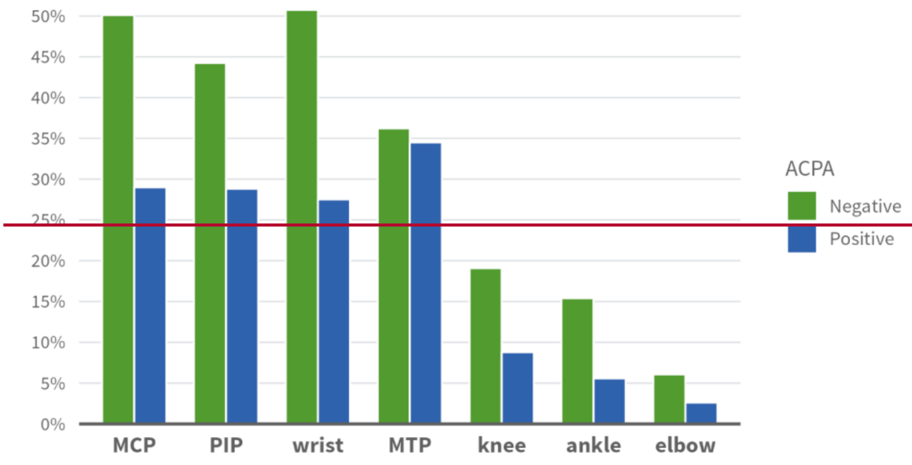

**Figure 1. Proportions of patients with symmetrical swelling by anatomical site in sero~~ACPA-~~positive and sero~~ACPA-~~negative patients, at the initial presentation of RA.**

## Disease activity measures

**Formatted:** Font: 16 pt

The median (IQR) CRP was 8 (3, 25) for all patients with incident RA, 7 (3, 21~~19~~) for sero~~ACPA-~~positive and 15 (5, 46~~0~~) for sero~~ACPA-~~negative patients (p<0.001). The median (IQR) ESR was 22~~1~~ (10, 39~~8~~) for all patients 20 (10, 37) for sero~~ACPA-~~positive and 26~~4~~ (12, 4~~2~~64) for sero~~ACPA-~~negative patients (p=0.006~~172~~, adjusted for age and sex).

A total of 53 % of all patients had normal CRP and 60~~1~~ % normal ESR. For sero~~ACPA-~~positive and negative patients, the proportions were 57~~8~~ % and 35~~9~~ % for CRP (p<0.001) and 63 % and 51~~5~~ % for ESR, respectively (p<0.001~~=0.014~~, adjusted for age and sex) (Table 2).

At the initial presentation, the median (IQR) DAS28-scores were 3.9 (3.1, 4.7) for all patients, 3.7 (3.0, 4.4) for sero~~ACPA-~~positive patients and 4.7~~5~~ (4~~3~~.0~~8~~, 5.7~~5~~) for sero~~ACPA-~~negative patients (p<0.001, adjusted for age and sex) (Table 2).

For Dr.global, the median (IQR) value was 40 (26, 56) for all patients, 39~~8~~ (25, 50) for sero~~ACPA~~ positive patients and 50~~49~~ (38~~3~~, 69~~2~~) for sero~~ACPA-~~negative patients (p<0.001, adjusted for age and sex) (Table 2).

## Patient reported outcomes

**Formatted:** Font: 16 pt

For pain, the median (IQR) VAS-score was 54 (30, 75) for all patients, 51 (30~~29~~, 74~~3~~) for sero~~ACPA~~ positive patients and 61~~0~~ (39~~6~~, 77~~8~~) for sero~~ACPA-~~negative patients (p<0.001, adjusted for age and sex). For fatigue, the corresponding numbers were 45 (18~~9~~, 70), 40 (14, 69) and 52 (3~~2~~1~~6~~, 73~~2~~) (p<0.001, adjusted for age and sex) and for PGA they were 50 (25, 67), 48~~6~~ (23~~8~~, 64~~9~~) and 53 (32, 70) (p<0.001, adjusted for age and sex) (Table 2).

The overall median (IQR) HAQ was 0.9 (0.5, 1.4) for all patients, 0.88 (0.38, 1.4) for sero~~ACPA-~~positive patients and 1.1 (0.75~~62~~, 1.5) for sero~~ACPA-n~~negative patients at the initial presentation (p<0.001, adjusted for age and sex) (Table 2).

# Initial medication

PPrednisolone was started included in the initial medication in in 82 % of seropositive patients and in 94 % of  seronegative patients (p<0.001). MTX as monotherapy was used in A total of 17 % of seropositive patients and forin 23 % of seronegative patients started with MTX monotherapy, 72% and 67% started MTX + csDMARD, 10% and 11% started csDMARD(s) only, while . The corresponding numbers for other csDMARDs as initial medication were 11 % and 10 % and bDMARDs and JAK-inhibitors were started initiated in only a few patientsprescribed only for byfor a few patients. GCs were started for 82 % of seropositive and 94 % of seronegative patients (Table 2).

Formatted: Font: 16 pt

Commented [TS2]: SIIRRÄ PREDNISOLON VIIMEISEKSI VIRKKEEKSI.

Commented [TS3]: EI VOIDA SANOA PRESCRIBED KOSKA EI VOI TIETÄÄ MITÄ NIILLE OLI MÄÄRÄTTY, TIEDETÄÄN VAIN MITÄ OLIVAT OSTANEET.

| Variable | Available data for all patients n, (%) | All | Available data for seroACPA positive patients n, (%) | SeroACPA positive | Available data for seroACPA negative patients n, (%) | SeroACPA negative | p-value |
|---|---|---|---|---|---|---|---|
| Number of patients, n | | 1816801 | | 1444294 (8072 %) | | 372507 (208 %) | |
| Median (IQR) SJC46 | 172306 (95 %) | 6 (3, 101) | 1371229 (95 %) | 5 (2, 9) | 352477 (954 %) | 109 (65, 154) | <0.001 |
| Median (IQR) TJC46 | 172306 (95 %) | 6 (3, 11) | 1371229 (95 %) | 5 (2, 109) | 352477 (954 %) | 109 (54, 187) | <0.001 |
| Proportions of patients | | | | | | | |

Formatted Table

| | | | | | | | |
|---|---|---|---|---|---|---|---|
| **with different SJC46 n, %**<br>≤2<br>3-5<br>≥6 | | 394388 (23 %)<br>43429 (25 %)<br>89488 (52 %) | | 36229 (267 %)<br>33943 (28 %)<br>629557 (465 %) | | 3259 (912 %)<br>5586 (1618 %)<br>265331 (7569 %) | <0.001<0.001 |
| **Proportions of patients with different TJC46 n, %**<br>≤2<br>3-5<br>≥6 | | 404398 (23 %)<br>43529 (25 %)<br>88479 (512 %) | | 35623 (26 %)<br>37843 (28 %)<br>637563 (46 %) | | 4675 (146 %)<br>5786 (168 %)<br>247316 (7066 %) | 0.014<0.001 |
| **Proportions of patients with symmetrical swelling by anatomical site n, %**<br>Any site<br>PIPs<br>MCPs<br>Wrists<br>Elbows<br>Knees | 162409 (89 %) | 1121403 (75 %)<br>5375 (33 %)<br>5684 (35 %)<br>550 (34 %) | 1285148 (89 %) | 906803 (710 %)<br>37531 (29 %)<br>37733 (29 %)<br>36516 (28 %) | 339461 (91 %) | 308400 (9187 %)<br>162204 (484 %)<br>191231 (560 %)<br>190234 (561 %) | <0.001<br><0.001<br><0.001<br><0.001<br><0.001<br><0.001<br><0.001<br>0.097547 |

| | | | | | | | |
|---|---|---|---|---|---|---|---|
| Ankles<br>MTPs | | 6058 (4 %)<br>19389 (12 %)<br>1365 (8 %)<br>5673 (35 %) | | 340 (3 %)<br>12401 (109 %)<br>7364 (6 %)<br>435396 (34 %) | | 2628 (86 %)<br>6988 (2019 %)<br>6371 (195 %)<br>13267 (396 %) | |
| **Median (IQR) CRP** | 171705 (95 %) | 8 (3, 25) | 1361222 (94 %) | 7 (3, 2109) | 356483 (965 %) | 195 (5, 460) | <0.001 |
| **Median (IQR) ESR** | 153623 (85 %) | 221 (10, 398) | 1221095 (85 %) | 20 (10, 37) | 315428 (854 %) | 264 (12, 464) | 0.006172 |
| **Proportions of patients with normal CRP or ESR n, %**<br>CRP<br>ESR | | 9030 (53 %)<br>9297 (601 %) | | 77710 (578 %)<br>768692 (63 %) | | 12690 (359 %)<br>191235 (515 %) | <0.001<0.001<br><0.0010.014 |
| **Median (IQR) DAS28** | 164429 (910 %) | 3.9 (3.1, 4.7) | 1308173 (91 %) | 3.7 (3.0, 4.54) | 336456 (90 %) | 4.75 (43.08, 5.75) | <0.001 |
| **Mean (SD) DAS28** | | 4.0 (1.2) | | 3.7 (1.1) | | 4.8 (1.3) | <0.001 |
| **Median (IQR) Dr.global** | 154328 (855 %) | 40 (26, 56) | 1233104 (855 %) | 398 (25, 50) | 310424 (834 %) | 5049 (383, 692) | <0.001 |
| **Median (IQR) PROs**<br>Pain<br>Fatigue | 159379 (88 %) | 54 (30, 75) | 1272141 (88 %) | 51 (3029, 743) | 321438 (86 %) | 610 (396, 778) | <0.001<br><0.001 |

**Formatted Table**

| | | | | | | | |
|---|---|---|---|---|---|---|---|
| PGA | 141806 (78 %) 159079 (88 %) | 45 (189, 70) 50 (25, 67) | 1128011 (78 %) 1273144 (88 %) | 40 (14, 69) 486 (238, 649) | 290395 (78 %) 317435 (856 %) | 52 (3126, 732) 543 (32, 70) | <0.001 |
| **Median (IQR) HAQ** | 143218 (79 %) | 0.90 (0.50, 1.40) | 1144024 (79 %) | 0.88 (0.38, 1.4) | 288394 (778 %) | 1.10 (0.7562, 1.50) | <0.001 |
| **Mean (SD) HAQ** | | 0.97 (0.69) | | 0.93 (0.69) | | 1.20 (0.66) | |
| **Initial Medication** | | | | | | | |
| | GC | | | | 1181 (82 %) | 348 (94 %) | <0.001 |
| **MTX monotherapy** | | | | 242 (17 %) | | 86 (23 %) | |
| **MTX and csDMARD** | | | | 1039 (72 %) | | 249 (67 %) | |
| **only csDMARD** | | | | 159 (11 %) | | 36 (10 %) | |
| **bDMARD** | | | | 3 (0 %) | | 1 (0 %) | |
| **JAK-inhibitor** | | | | 1 (0 %) | | | |
| **GC** | | | | 1181 (82 %) | | 348 (94 %) | |

Commented [TS4]: tämä alimmaiseksi ja jätä tästäkin p pois

**Table 2. Clinical data of patients with RA at the initial presentation, by serological statusn, by ACPA status.**

Commented [TS5]: KORJAA

## Effect of diagnostic delay uration of symptoms on the clinical picture

Formatted: Font: 16 pt

Between patient groups by diagnostic delayduration of symptoms, there were notable differences in terms of ACPA-seroACPA-positivity, DAS28, ESR, pain and HAQ. For other variables, such as SJC46, TJC46, CRP, Dr.global, fatigue and PGA, the differences between groups were minor and mostly statistically not significant (S2 Table 4).

*SeroACPAACPA--positivity* was highest the most prevalent with a proportion of 7776 % in group 3 with a duration of symptoms of >6 months, compared to 70 % and 70 %69 % in other groups (p=<0.045001, adjusted for age and sex). There were similar results also for seropositivity (78 %, 77 % and 82 % in groups 1,2 and 3, p=0.115)- and RF-positivity (68 %, 67 % and 71 % in groups 1, 2 and 3, p=0.324) (S2 Table 4).

Commented [TS6]: sero what

*Disease activity.* The median (IQR) DAS28 was 4.1 (3.3, 5.10) in group 1, 4.0 (3.2, 4.8) in group 2 and 3.8 (3.10, 4.5) in group 3 (p=0.0168, adjusted for age and sex). The median (IQR) ESR was 276 (12, 44) in group 1, 2019 (10, 36) in group 2 and 187 (9, 343) in group 3 (p<=0.0018, adjusted for age and sex) and the proportions of patients with a normal level of ESR were 55 %, 61 % and 667 % in the same groups, respectively (p=0.0466, adjusted for age and sex) (S2 Table 4).

*PROs.* For median (IQR) pain, the values in groups 1, 2 and 3 were 62 (35, 78), 52 (30, 72) and 50 (26, 70) (p<0.001, adjusted for age and sex). For median (IQR) HAQ, the values in the corresponding groups were 1.10 (0.50, 1.60), 0.9 (0.50, 1.40) and 0.75 (0.38, 1.20) (p=0.0023, adjusted for age and sex) (S2 Table 4).

## Clinical variables between ACPA-positive andbut RF-negative and RF-positive butand ACPA-negative patients

Formatted: Font: 16 pt

A total of 163 (11 % of seropositive patients) were only ACPA-positive and 118 (8 % of seropositive patients) were only RF-positive. The mean (SD) age was 53 (17) for solely ACPA- and 62 (14) for solely RF-positive patients (p<0.001) and the duration of symptoms for the same groups were 6 (3, 13) and 4 (2, 7) (p=0.222). The median (IQR) DAS28 was 3.6 (2.8, 4.2) for only ACPA-positive patients and 4.2 (3.5, 5.0) for only RF-positive patients (p<0.001). The same numbers for median (IQR) HAQ were 0.62 (0.3, 1.1) and 1.1 (0.5, 1.5) (p=0.033). Besides DAS28 and HAQ, there were no statistically

significant differences, though all of the clinical variables were higher for only RF-positive patients (Table 3).

| Variable | Available data for ACPA-positive patients | ACPA-positive | Available data for RF-positive patients | RF-positive | p-value |
|---|---|---|---|---|---|
| n, % of seropositive patients | | 163 (11 %) | | 118 (8 %) | |
| Age | | 53 (17) | | 62 (14) | <0.001 |
| Sex | | 112 (69 %) | | 84 (71 %) | |
| Duration of symptoms | 124 (76 %) | 6 (3, 13) | 87 (74 %) | 4 (2, 7) | 0.222 |
| Median (IQR) SJC46 | 156 (96 %) | 4 (2, 8) | 113 (96 %) | 6 (3, 10) | 0.193 |
| Median (IQR) TJC46 | 156 (96 %) | 4 (2, 7) | 113 (96 %) | 7 (3, 12) | 0.056 |
| Median (IQR) CRP | 152 (93 %) | 5 (3, 13) | 111 (94 %) | 9 (3, 26) | 0.458 |
| Median (IQR) ESR | 137 (84 %) | 13 (7, 28) | 99 (84 %) | 20 (10, 35) | 0.780 |
| Median (IQR) DAS28 | 148 (91 %) | 3.6 (2.8, 4.2) | 108 (92 %) | 4.2 (3.5, 5.0) | <0.001 |
| Median (IQR) Dr.global | 137 (84 %) | 35 (25, 50) | 103 (87 %) | 40 (27, 50) | 0.194 |
| Median (IQR) PROs Pain | | | | | |

Commented [TS7]: TARKISTA TAULUJEN NUMEROT – KOMMENTEISSA KIRJOITAT ESIM S2, TÄMÄ TAULU ON 3 – ON VÄHÄN EPÄJOHDONMUKAISUUTTA

Formatted: Font: Not Bold

Formatted Table

| | | | | | |
|---|---|---|---|---|---|
| **Fatigue**<br>**PGA** | 146 (90 %)<br>131 (80 %)<br>146 (90 %) | 50 (27, 75)<br>50 (20, 70)<br>50 (25, 64) | 104 (88 %)<br>91 (77 %)<br>103 (87 %) | 58 (33, 77)<br>46 (18, 74)<br>51 (32, 70) | 0.145<br>0.767<br>0.402 |
| **Median (IQR) HAQ** | 134 (82 %) | 0.62 (0.3, 1.1) | 93 (79 %) | 1.1 (0.5, 1.5) | 0.033 |

**Table 3. Clinical data of patients that were only ACPA- or RF-positive.**

**Formatted:** Font: 12 pt

# Discussion

**Formatted:** Font: 18 pt

Our main observation was that early RA is still mainly presented as symmetric arthritis, as three out of four patients had symmetrical swelling in any of the anatomical sites of the 1987 criteria at the initial presentation (1). Overall, the wrists were the most commonly swollen joints by a significant margin (S1 Table~~ 3~~). Symmetrical swelling was detected more often overall and at all sites in ACPA negative patients. This was an expected finding since more swollen joints are required to fulfill the criteria for seronegative RA (5). Sero~~ACPA ~~negative patients had also higher SJC46 at the initial presentation (median 5 *versus* 10~~9~~). Interestingly, the presence of symmetrical swelling was only slightly higher for sero~~ACPA ~~negative patients in the MTPs (34 % *versus* 39~~6~~ %, p=0.09~~54~~7) (Table 2) despite higher SJC46.

*Clinical measures and medication*. In terms of clinical data, sero~~ACPA ~~negative RA presented significantly higher activity by almost all variables used in the study (Table 2). Similar differences

were found between solely ACPA-positive and solely RF-positive patients, though most of the findings weren't statistically significant (Table 3). In ESR, the difference was only minor between seropositive and seronegative patients (20 *versus* 24, p=0.172) (Table 2). Sero~~ACPA-~~positive patients were more likely to fulfill the ACR/EULAR 2010 criteria (82 % *versus* 78) at the time of diagnosis. Patients with sero~~ACPA-~~positive RA were also current smokers more often, (21 % *versus* 12 % current smokers) (Table 1), as smoking is a known risk-factor for ~~ACPA-~~ACPA-positive RA (24). In terms of medication, GC's and MTX as monotherapy was used more frequently in patients that were diagnosed with seronegative RA. The more prevalent use of GCs~~, which~~ indicates that some of the diagnoses~~m~~ might have been actually polymyalgia rheumaticas, as our previous studies have suggested (10,11).

*Comparison to other studies.* The results between sero~~ACPA-~~positive and negative RA were somewhat contradictory compared to earlier research, a change likely caused by aiming at earlier diagnosis in accordance with the 2010 classification criteria. A French study of 354 patients diagnosed between 2002 and 2005 showed that seropositive patients had higher average DAS28 (5.3 *versus* 5.0) and HAQ (1.0 *versus* 0.9) than seronegative patients, but similar joint counts and VAS-values of pain (12). In our study, the variables were significantly higher for sero~~ACPA-~~negative RA (mean DAS28 of 4.8~~6~~ *versus* 3.7 and 1.2~~1~~ *versus* 0.93~~1~~ for HAQ~~, data not shown~~). However, the populations are not directly comparable, as the study had ~~17,5 % RF-positive and ACPA-negative patients in the seropositive group and~~ slightly different threshold values for ACPA and RF.

A Danish study of 198 ~~(94.4 % ACPA-positive)~~ newly diagnosed seropositive and 36 seronegative patients diagnosed in 2010 to 2013 showed lower median SJC44 (8 *versus* 17), Dr.global (39 *versus* 49) and DAS28 (3.4 *versus* 3.9), but similar PROs at the initial presentation in ACPA positive *versus* negative patients (25). On the contrary, our population showed significantly lower PROs at the initial presentation in ~~ACPA-~~seropositive *versus* sero~~ACPA-~~negative patients (51 *versus* 61~~0~~ for pain, 40~~0~~ *versus* 52 for fatigue and 48~~6~~ *versus* 54~~3~~ for PGA) (Table 2), in addition to other clinical variables.

In terms of duration of symptoms, ~~diagnostic delay,~~ a previous study of patients diagnosed in 2007-2012 in Canada showed a ~~delay~~ duration of symptoms of 6.5 months for patients with ~~ACPA-~~seropositive RA and 5.4 months for patients with seronegative RA (26). This is in line with our results, where the ~~-~~median ~~diagnostic delay~~duration of symptoms was five months for ~~ACPA-~~seropositive and four months for ~~ACPA-~~seronegative RA (Table 1).

**Commented [LW8]:** Viitteitä johonkin vai pelkka pohdinta???

*The effect of ~~diagnostic delay~~duration of symptoms on the initial presentation.* The overall median SJC46 was the same in all groups by ~~diagnostic delay~~duration of symptoms (median 6 joints) (S2 Table ~~2~~) and median DAS28 was higher in the group of patients with a delay of < 3 months compared to patients with a delay of >6 months (4.1 *versus* 3.8, p=0.016~~8~~, adjusted for age and sex). Interestingly, patients with a delay of <3 and 3-6 months were also older than in the group with a delay of >6 months~~, though not statistically significantly~~ (mean age of 61 and 58 in groups with a delay of <3 and 3-6 months and 56 in the group with a delay of >6 months, p<0.001~~=0.581~~) (S2 Table 4). It has been shown that older patients utilize health care services more frequently, which might explain the finding (27). The overall median CRP and ESR were higher in patients who were diagnosed earlier (11 in the group with a delay of <3 months, *versus* 8 and 7 in groups with a delay of 3-6 months and >6 months for CRP) and (27~~6~~ *versus* 20 and 18~~7~~ for ESR in the same groups, correspondingly). High laboratory values might have influenced towards early referrals, which in turn lowers the ~~diagnostic delay~~duration of symptoms. In addition, the median VAS-pain and HAQ were also significantly higher in patients who were diagnosed earlier (62 in the group with a delay of <3 months *versus* 52 and 50 in groups with 3-6 and >6 months, for pain) and (1.10 *versus* 0.90 and 0.75 for the same groups for HAQ), but ACPA was positive less frequently (77~~6~~ % in the group with a delay of >6 months *versus* 70~~69~~ % and 70~~69~~ % in other groups, p=0.045) (S2 Table ~~4~~), which indicates that patients experiencing more severe symptoms and limitations in physical activity might seek health care services earlier. ACPA-positivity isn't necessarily associated with the severity of symptoms. A previous Polish study has shown that the second most important factor for the ~~diagnostic delay~~duration of symptoms of RA is patients' conviction that the condition will resolve on its own (28).

# Strengths and limitations

**Formatted:** Font: 18 pt

*The main strength* of this study was its large patient population from almost all health care regions, in Finland and that patient data was documented recently allowing an accurate presentation of the current clinical picture.

*Weaknesses.* Although all health care regions are included in the quality register, at the time that was defined as the focus period, not all regions actively recorded clinical data. Therefore, probably not all patients with incident RA could be included. Furthermore, although not recommended, patients may still be diagnosed along with receiving treatment initiation in private practice, which are not involved in the quality register, yet.

One of the weaknesses in register studies is missing data, although the completeness was as high as ≥95% for joint counts and ≥78 % for PROs.

# Conclusions

**Formatted:** Font: 18 pt

Our results indicate that the initial presentation of early RA is still mostly symmetric sero~~ACPA~~ positive arthritis. Furthermore, we found that sero~~ACPA~~ negative RA has significantly higher inflammatory activity and disease burden at the initial presentation by several clinical variables, compared to sero~~ACPA~~ positive cases. However, 25 % of patients in the current study didn't have symmetrical swelling at the initial presentation, which is why all patients with clinical joint inflammation of unknown reason ought to be tested for ACPA and RF and referred to a rheumatology unit if ACPA and/or RF is~~ACPA they are~~ positive. Our observations are encouraging in terms that patients experiencing more severe pain and decrease in functional ability might seek health care services earlier. We also found that ACPA-~~ACPA~~-positivity is not necessarily associated with the severity of symptoms.

~~**Acknowledgements**~~

We would like to thank The Finnish Psoriasis Association, The Finnish Society for Rheumatology, and Amgen. The author also acknowledges the Research Committee of the Kuopio University Hospital Catchment Area for the State Research Funding (project 5041730, Kuopio, Finland).

# References:

1. Arnett F. Classification criteria: The 1987 American Rheumatism Association revised criteria for the classification of rheumatoid arthritis. CPD Rheumatol. 1999;1(1):13–4.
2. Bukhari M, Lunt M, Harrison BJ, Scott DGI, Symmons DPM, Silman AJ. Erosions in inflammatory polyarthritis are symmetrical regardless of rheumatoid factor status: Results from a primary care-based inception cohort of patients. Rheumatology. 2002;41(3):246–52.
3. Zangger P, Keystone EC, Bogoch ER. Asymmetry of small joint involvement in rheumatoid arthritis: Prevalence and tendency towards symmetry over time. Jt Bone Spine. 2005;72(3):241–7.
4. Sanchez-Castillo JA, Reinoso-Cobo A, Gijon-Nogueron G, Caliz-Caliz R, Exposito-Ruiz M, Ramos-Petersen L, et al. Symmetry criterion for patients with rheumatoid arthritis of the foot: A cross-sectional study. Int J Environ Res Public Health. 2021;18(7).
5. Aletaha D, Neogi T, Silman AJ, Funovits J, Felson DT, Bingham CO, et al. 2010 Rheumatoid arthritis classification criteria: An American College of Rheumatology/European League Against Rheumatism collaborative initiative. Arthritis Rheum. 2010;62(9):2569–81.
6. Van Der Helm-Van Mil AHM, Zink A. What is rheumatoid arthritis? Considering consequences of changed classification criteria. Ann Rheum Dis. 2017;76(2):315–7.
7. Raychaudhuri S. NIH Public Access. Curr Opin Rheumatol. 2010;22(2):109–18.
8. Chang K, Yang SM, Kim SH, Han KH, Park SJ, Shin J Il. Smoking and rheumatoid arthritis. Int J Mol Sci. 2014;15(12):22279–95.
9. Asikainen J, Nikiphorou E, Kaarela K, Lindqvist E, Häkkinen A, Kautiainen H, et al. Is long-term radiographic joint damage different between men and women? Prospective longitudinal data analysis of four early RA cohorts with greater than 15 years of follow-up.

Formatted: Font: 18 pt

Formatted: English (United States)

Formatted: Swedish (Sweden)

Formatted: English (United States)

Formatted: English (United States)

Vol. 34, Clinical and Experimental Rheumatology. 2016.

10. Paalanen K, Rannio K, Rannio T, Asikainen J, Hannonen P, Sokka T. Does early seronegative arthritis develop into rheumatoid arthritis? A 10-year observational study. Clin Exp Rheumatol. 2019;37(1):37–43.

11. Paalanen K, Puolakka K, Nikiphorou E, Hannonen P, Sokka T. Is seronegative rheumatoid arthritis true rheumatoid arthritis? A nationwide cohort study. Rheumatol (United Kingdom). 2021;60(5):2391–5.

12. Mouterde G, Rincheval N, Lukas C, Daien C, Saraux A, Dieudé P, et al. Outcome of patients with early arthritis without rheumatoid factor and ACPA and predictors of rheumatoid arthritis in the ESPOIR cohort. Arthritis Res Ther. 2019;21(1):1–9.

13. Luukkainen R, Kajander A, Isomaki H. Treatment of rheumatoid arthritis. Vol. 2, British Medical Journal. 1978. p. 1501.

14. Emery P, Salmon M. Early rheumatoid arthritis: Time to aim for remission? Ann Rheum Dis. 1995;54(12):944–7.

15. Smolen JS, Aletaha D, Bijlsma JWJ, Breedveld FC, Boumpas D, Burmester G, et al. Treating rheumatoid arthritis to target: Recommendations of an international task force. Ann Rheum Dis. 2010;69(4):631–7.

16. Gremese E, Salaffi F, Bosello SL, Ciapetti A, Bobbio-Pallavicini F, Caporali R, et al. Very early rheumatoid arthritis as a predictor of remission: A multicentre real life prospective study. Ann Rheum Dis. 2013;72(6):858–62.

17. Quinn MA, Emery P. Window of opportunity in early rheumatoid arthritis: Possibility of altering the disease process with early intervention. Clin Exp Rheumatol. 2003;21(5 SUPPL. 31).

18. Sørensen J, Hetland ML. Diagnostic delay in patients with rheumatoid arthritis, psoriatic arthritis and ankylosing spondylitis: Results from the Danish nationwide DANBIO registry. Ann Rheum Dis. 2015;74(3):1–7.

19. Van Der Linden MPM, Le Cessie S, Raza K, Van Der Woude D, Knevel R, Huizinga TWJ, et al. Long-term impact of delay in assessment of patients with early arthritis. Arthritis Rheum.

2010;62(12):3537–46.

20. Mankia K, Nam J, Emery P. Identifying arthralgia suspicious for progression to rheumatoid arthritis. Ann Rheum Dis. 2017;76(6):e14.

21. Prevoo MLL, Van'T Hof MA, Kuper HH, Van Leeuwen MA, Van De Putte LBA, Van Riel PLCM. Modified disease activity scores that include twenty-eight-joint counts development and validation in a prospective longitudinal study of patients with rheumatoid arthritis. Arthritis Rheum. 1995;38(1):44–8.

22. Krishnan E, Sokka T, Häkkinen A, Hubert H, Hannonen P. Normative Values for the Health Assessment Questionnaire Disability Index: Benchmarking Disability in the General Population. Arthritis Rheum. 2004;50(3):953–60.

23. Sokka T, Toloza S, Cutolo M, Kautiainen H, Makinen H, Gogus F, et al. Women, men, and rheumatoid arthritis: Analyses of disease activity, disease characteristics, and treatments in the QUEST-RA Study. Arthritis Res Ther. 2009;11(1).

24. Hedström AK, Stawiarz L, Klareskog L, Alfredsson L. Smoking and susceptibility to rheumatoid arthritis in a Swedish population-based case–control study. Eur J Epidemiol ~~[Internet]~~. 2018;33(4):415–23. ~~Available from: https://doi.org/10.1007/s10654-018-0360-5~~

25. Nordberg LB, Lillegraven S, Lie E, Aga AB, Olsen IC, Hammer HB, et al. Patients with seronegative RA have more inflammatory activity compared with patients with seropositive RA in an inception cohort of DMARD-naïve patients classified according to the 2010 ACR/EULAR criteria. Ann Rheum Dis. 2017;76(2):341–5.

26. Barra L, Pope JE, Orav JE, Boire G, Haraoui B, Hitchon C, et al. Prognosis of seronegative patients in a large prospective cohort of patients with early inflammatory arthritis. J Rheumatol. 2014;41(12):2361–9.

27. Nie JX, Wang L, Tracy CS, Moineddin R, Upshur RE. Health care service utilization among the elderly: Findings from the Study to Understand the Chronic Condition Experience of the Elderly and the Disabled (SUCCEED project). J Eval Clin Pract. 2008;14(6):1044–9.

28. Raciborski F, Kłak A, Kwiatkowska B, Batko B, Sochocka-Bykowska M, Zoń-Giebel A, et al. Diagnostic delays in rheumatic diseases with associated arthritis. Reumatologia.


**Formatted:** Swedish (Sweden)

**Formatted:** English (United States)

**Formatted:** English (United States)

**Formatted:** Swedish (Sweden)

**Formatted:** English (United States)

**Formatted:** Swedish (Sweden)

**Formatted:** English (United States)

2017;55(4):169–76.

**Lähettäjä:** Lauri Weman <lauriweman@gmail.com>
**Lähetetty:** keskiviikko 25. tammikuuta 2023 18.03
**Vastaanottaja:** Sokka-Isler Tuulikki <tuulikki.sokka-isler@ksshp.fi>
**Aihe:** Fwd: PLOS ONE Decision: Revision required [PONE-D-22-32344] - [EMID:efbd7df372adebd2]

---------- Forwarded message ---------
Lähettäjä: **PLOS ONE** <em@editorialmanager.com>
Date: ke 25. tammik. 2023 klo 18.01
Subject: PLOS ONE Decision: Revision required [PONE-D-22-32344] - [EMID:efbd7df372adebd2]
To: Lauri Weman <lauriweman@gmail.com>

CC: sumar@uic.edu, sadiq36@gmail.com

PONE-D-22-32344
Initial presentation of early rheumatoid arthritis
PLOS ONE

Dear Dr. Weman,

Thank you for submitting your manuscript to PLOS ONE. After careful consideration, we feel that it has merit but does not fully meet PLOS ONE's publication criteria as it currently stands. Therefore, we invite you to submit a revised version of the manuscript that addresses the points raised during the review process.

Please submit your revised manuscript by Mar 11 2023 11:59PM. If you will need more time than this to complete your revisions, please reply to this message or contact the journal office at plosone@plos.org. When you're ready to submit your revision, log on to https://www.editorialmanager.com/pone/ and select the 'Submissions Needing Revision' folder to locate your manuscript file.

Please include the following items when submitting your revised manuscript:

- A rebuttal letter that responds to each point raised by the academic editor and reviewer(s). You should upload this letter as a separate file labeled 'Response to Reviewers'.
- A marked-up copy of your manuscript that highlights changes made to the original version. You should upload this as a separate file labeled 'Revised Manuscript with Track Changes'.
- An unmarked version of your revised paper without tracked changes. You should upload this as a separate file labeled 'Manuscript'.

If you would like to make changes to your financial disclosure, please include your updated statement in your cover letter. Guidelines for resubmitting your figure files are

available below the reviewer comments at the end of this letter.

If applicable, we recommend that you deposit your laboratory protocols in protocols.io to enhance the reproducibility of your results. Protocols.io assigns your protocol its own identifier (DOI) so that it can be cited independently in the future. For instructions see: https://journals.plos.org/plosone/s/submission-guidelines#loc-laboratory-protocols. Additionally, PLOS ONE offers an option for publishing peer-reviewed Lab Protocol articles, which describe protocols hosted on protocols.io. Read more information on sharing protocols at https://plos.org/protocols?utm_medium=editorial-email&utm_source=authorletters&utm_campaign=protocols.

We look forward to receiving your revised manuscript.

Kind regards,

Sadiq Umar
Academic Editor
PLOS ONE

Journal Requirements:

When submitting your revision, we need you to address these additional requirements.

1. Please ensure that your manuscript meets PLOS ONE's style requirements, including those for file naming. The PLOS ONE style templates can be found at https://journals.plos.org/plosone/s/file?id=wjVg/PLOSOne_formatting_sample_main_body.pdf and https://journals.plos.org/plosone/s/file?id=ba62/PLOSOne_formatting_sample_title_authors_affiliations.pdf

2. We note that the grant information you provided in the ‘Funding Information’ and ‘Financial Disclosure’ sections do not match.

When you resubmit, please ensure that you provide the correct grant numbers for the awards you received for your study in the ‘Funding Information’ section.

3. Thank you for stating the following in the Acknowledgments Section of your manuscript:

"We would like to thank The Finnish Psoriasis Association, The Finnish Society for Rheumatology,and Amgen. The author also acknowledges the Research Committee of the Kuopio University Hospital Catchment Area for the State Research Funding (project 5041730, Kuopio, Finland)."

We note that you have provided funding information that is not currently declared in your Funding Statement. However, funding information should not appear in the

Acknowledgments section or other areas of your manuscript. We will only publish funding information present in the Funding Statement section of the online submission form.
Please remove any funding-related text from the manuscript and let us know how you would like to update your Funding Statement. Currently, your Funding Statement reads as follows:
"NO"

Please include your amended statements within your cover letter; we will change the online submission form on your behalf.

4. Thank you for stating the following in your Competing Interests section:
"NO"
Please complete your Competing Interests on the online submission form to state any Competing Interests. If you have no competing interests, please state "The authors have declared that no competing interests exist.", as detailed online in our guide for authors at http://journals.plos.org/plosone/s/submit-now
This information should be included in your cover letter; we will change the online submission form on your behalf.

5. In your Data Availability statement, you have not specified where the minimal data set underlying the results described in your manuscript can be found. PLOS defines a study's minimal data set as the underlying data used to reach the conclusions drawn in the manuscript and any additional data required to replicate the reported study findings in their entirety. All PLOS journals require that the minimal data set be made fully available. For more information about our data policy, please see http://journals.plos.org/plosone/s/data-availability.

"Upon re-submitting your revised manuscript, please upload your study's minimal underlying data set as either Supporting Information files or to a stable, public repository and include the relevant URLs, DOIs, or accession numbers within your revised cover letter. For a list of acceptable repositories, please see http://journals.plos.org/plosone/s/data-availability#loc-recommended-repositories. Any potentially identifying patient information must be fully anonymized.

Important: If there are ethical or legal restrictions to sharing your data publicly, please explain these restrictions in detail. Please see our guidelines for more information on what we consider unacceptable restrictions to publicly sharing data: http://journals.plos.org/plosone/s/data-availability#loc-unacceptable-data-access-restrictions. Note that it is not acceptable for the authors to be the sole named individuals responsible for ensuring data access.

We will update your Data Availability statement to reflect the information you provide in your cover letter.

6. PLOS requires an ORCID iD for the corresponding author in Editorial Manager on papers submitted after December 6th, 2016. Please ensure that you have an ORCID iD and that it is validated in Editorial Manager. To do this, go to 'Update my Information' (in the upper left-hand corner of the main menu), and click on the Fetch/Validate link next to the ORCID field. This will take you to the ORCID site and allow you to create a new iD or authenticate a pre-existing iD in Editorial Manager. Please see the following video for instructions on linking an ORCID iD to your Editorial Manager account: https://www.youtube.com/watch?v=_xcclfuvtxQ

7. Please ensure that you include a title page within your main document. You should list all authors and all affiliations as per our author instructions and clearly indicate the corresponding author.

8. We note that you have included the phrase "data not shown" in your manuscript. Unfortunately, this does not meet our data sharing requirements. PLOS does not permit references to inaccessible data. We require that authors provide all relevant data within the paper, Supporting Information files, or in an acceptable, public repository. Please add a citation to support this phrase or upload the data that corresponds with these findings to a stable repository (such as Figshare or Dryad) and provide and URLs, DOIs, or accession numbers that may be used to access these data. Or, if the data are not a core part of the research being presented in your study, we ask that you remove the phrase that refers to these data.

9. Please include your full ethics statement in the 'Methods' section of your manuscript file. In your statement, please include the full name of the IRB or ethics committee who approved or waived your study, as well as whether or not you obtained informed written or verbal consent. If consent was waived for your study, please include this information in your statement as well.

10. Please include a copy of Table 3 and 4 which you refer to in your text on page 10 and 15.

11. Please include captions for your Supporting Information files at the end of your manuscript, and update any in-text citations to match accordingly. Please see our Supporting Information guidelines for more information: http://journals.plos.org/plosone/s/supporting-information.

[Note: HTML markup is below. Please do not edit.]

Reviewers' comments:

Reviewer's Responses to Questions

**Comments to the Author**

1. Is the manuscript technically sound, and do the data support the conclusions?

The manuscript must describe a technically sound piece of scientific research with data that supports the conclusions. Experiments must have been conducted rigorously, with appropriate controls, replication, and sample sizes. The conclusions must be drawn appropriately based on the data presented.

Reviewer #1: Yes

Reviewer #2: Yes

---

2. Has the statistical analysis been performed appropriately and rigorously?

Reviewer #1: Yes

Reviewer #2: Yes

---

3. Have the authors made all data underlying the findings in their manuscript fully available?

The PLOS Data policy requires authors to make all data underlying the findings described in their manuscript fully available without restriction, with rare exception (please refer to the Data Availability Statement in the manuscript PDF file). The data should be provided as part of the manuscript or its supporting information, or deposited to a public repository. For example, in addition to summary statistics, the data points behind means, medians and variance measures should be available. If there are restrictions on publicly sharing data—e.g. participant privacy or use of data from a third party—those must be specified.

Reviewer #1: Yes

Reviewer #2: No

---

4. Is the manuscript presented in an intelligible fashion and written in standard English?

PLOS ONE does not copyedit accepted manuscripts, so the language in submitted articles must be clear, correct, and unambiguous. Any typographical or grammatical errors should be corrected at revision, so please note any specific errors here.

Reviewer #1: Yes

Reviewer #2: Yes

---

5. Review Comments to the Author

Please use the space provided to explain your answers to the questions above. You may also include additional comments for the author, including concerns about dual publication, research ethics, or publication ethics. (Please upload your review as an attachment if it exceeds 20,000 characters)

**Reviewer #1:** The authors describe the initial presentation of early rheumatoid arthritis patients in Finland, using national databases. The result may be of interesting to readers, however, I have a couple of concerns. Specific comments are listed below:

1. The authors compare clinical items between ACPA-positive RA patients and negative patients, this is a bit confusing because so called seropositive RA includes RF+/ACPA- patients. This study may include such patients in ACPA negative RA patients, which shows a bit different nuance compared to 'seronegative' RA. More explanation of this may be needed to clarify the characteristics of patient group. I also be interested the clinical feature of ACPA-/RF+ RA patients (can be difficult to investigate, though).

**Response:** According to the Reviewers's suggestion, we now present the data for seropositive (ACPA or RF positive) vs. seronegative (double negative) patients. In addition, we present a separate table for 163 ACPA+/RF- patients vs. 118 ACPA-/RF+ patients.

2. We routinely use EULAR/ACR criteria for RA diagnosis in daily clinical practice, and due to the setting of scoring system, it is understandable ACPA negative (seronegative??) RA patients show more swollen joints (symmetrical) or high CRP levels. Therefore, it is natural to predict the results in this study. In other words, what is the most interesting findings in this study, compared to previous findings? Please address more clearly.

**Response:** We have shown (Weman&Sokka, Duodecim 2021, in Finnish) in an analysis from our own clinic, that RA does not present as polyarthritis but the initial presentation currently is arthritis in 1-2 joints in more than 50% of patients + positive ACPA. This is what he hypothesized concerning patients from the whole country. Unfortunately this is not true and we have to report that in Finland, the initial presentation of RA is still

symmetric polyarthritis. - However, our group felt that we cannot open this up in the manuscript but merely report the findings.

3. This study dose not describe initial therapy after diagnosis of RA. Are there any differences (if can)?

**Response:** We added the analyses of the initial medication.

**Reviewer #2**: The authors studied the initial clinical presentation of alarge number of RA patients using national databases from Finland, with a particular interest in the impact of the presence of ACPA on the clinical presentation. A fair concern related to the objective of this study is that patients that do not present with symmetric polyarthritis could be having their diagnosis delayed. They concluded that incident RA presents mainly as symmetric arthritis, with higher disease severity in ACPA-negative patients. . This is not surprising, since as the authors themselves mentioned, the current classification criteria gives weight to seropositivity and, in negative patients, to the number of joints. On the other hand, it was surprising that ACAP-pos patients had a longer delay in diagnosis than ACPA-neg, when we know that ACPA is correlated with disease severity. For me, this suggests that there may be present a recruitment bias in this study: the ACPA-neg patients recruited could be representative of a more severe spectrum, while patients with milder forms are being left undiagnosed as RA, perhaps receiving other labels, such as "undifferentiated arthritis". The authors should discuss that possibility since it can have a great bearing on the main conclusions of the study.

**Response:** This result that ACPA positive patients had a longer delay in diagnosis vs. ACPA negative was also surprising to us. It is possible that the double seronegative group included patients who should have been labelled as polymyalgia rheumatica. However through this database we cannot investigate this further. We included this in discussion.

Other issues/recommendations:

1. The authors should comment on the criteria for the request of ACPA, since about 11% of incident RA patients were not included in the analysis because they did not have ACPA available in their records. Could all these patients be RF positive, with high disease activity and, therefore, ACPA was forsaken because of obvious diagnosis? This could alter some of the conclusions that ACPA-neg patients have higher disease activity and shorter diagnostic delay.

**Response**: Practically all patients with the suspicion of RA are being tested for ACPA and RF in Finland. The reason for the missing ACPA/RF values is simple: there was a delay in the programming of the automatic data transfer from the laboratory databases and therefore, the ACPA/RF values had to available from the clinical databases where the values had to be entered by hand. If the “hand work” was missing, the values were missing in these analyses. However, in the future, all laboratory data taken will be

available in the national rheumatology database, as soon as the automatic laboratory transfer will be ready.

The reviewer asks whether all these patients who had missing ACPA were RF positive and therefore ACPA would not even had been taken – and had high disease activity. This was not the case. The 216 patients that weren't included in the analyses had a median duration of symptoms of 6 months, being longer compared to the patients who were ACPA-tested. The median DAS28-score was 3.6 for these 216 patients, whereas the median DAS28 was 3.7 for ACPA-positive patients and 4.5 for ACPA-negative patients.

2. The variables in Methods should be presented in full sentences, with better descriptions, and not by hyphenated-items. Particular confusing are the variables used to represent the duration of disease: "duration of symptoms", "fulfillment of ACR/EULAR 2010-criteria" and "diagnostic delay". These terms should be standardized throughout the text.

**Response:** We have now explained the two variables with more detail. The time between first symptoms and the diagnosis of RA is now referred only as ''duration of symptoms''.

-duration of symptoms: time between the first symptoms of RA and the diagnosis of RA

-Fulfillment of the ACR/EULAR 2010 criteria: clinical diagnosis of RA by rheumatologist who counts the items in the ACR/EULAR 2010 criteria, if score adds up to 6 or more points, the ACR/EULAR 2010 criteria are met, when also other reasons that can explain the condition are excluded.

3. In terms of disease duration, the median (IQR) of 4 (2,10) months is very short, indicating that most (if not all) patients have relatively early (< 1y) disease. This should be discussed: is it true that very few patients in Finland are being diagnosed with longer than 1 year disease duration? Perhaps the ranges of disease duration could be presented, particularly for the groups by diagnosis delay.

**Response:** We present the range of disease duration in S2 table.

Concerning the "short" duration of symptoms before diagnosis we want to explain the setting of the Finnish rheumatology care:

Historically, there has been a strong vision to get RA under control in Finland. The Rheumatism Foundation Hospital was established in 1951 in Heinola and it also served as the national center of research and education in rheumatic diseases. Their vision was well described in a letter published in Br Med Journal in 1978, as a response to critique to their enthusiasm concerning intra muscular gold in early RA: "Gold treatment ought to be started before erosions…. We are treating not only the actual inflammation of the joints but also the quality of the patient's life for many decades in the future". (Luukkainen R, Kajander A, Isomäki H. Br Med J 1978; 2:1501). This was the Finnish manifest of T2T, 30 years before its

time. The FIN-RAco study in the 1990's increased the awareness of the urgency to make the diagnosis of RA early. The availability of the ACPA test in early 2000's helped to recognize true seropositive cases of RA.

Education. There are only five medical schools in Finland; rheumatology educators are a small team and it has been clear for long that every medical student has to know, how to suspect early RA, test ACPA, and make a referral. Furthermore, national "general practice guidelines" are available in the internet (since 20 or so years, and updated regularly) to all health care professionals, e.g. what to do if a patient has a swollen joint – or a suspicion of a swollen joint.

Structure of health care is based on shared care between general health care and specialty care. Diagnosis of RA belongs to the rheumatology multidisciplinary clinic, with specialized nurses for patient education. The "early RA treatment path" describes the follow-up of the patient and the role of general health care and the rheumatology clinic.

Therefore, it is not surprising to us that the median duration of symptoms before diagnosis was 4 months nationwide. In fact, it should be shorter as we discuss in the manuscript.

4. Tables 3 and 4 are supplementary - this should be indicated in the main text when they are referred to.

**Response:**They are now referred as S1 table and S2 table, according to the manuscript guidelines.

---

6. PLOS authors have the option to publish the peer review history of their article (what does this mean?). If published, this will include your full peer review and any attached files.

If you choose "no", your identity will remain anonymous but your review may still be made public.

**Do you want your identity to be public for this peer review?** For information about this choice, including consent withdrawal, please see our Privacy Policy.

Reviewer #1: No

Reviewer #2: No

---

[NOTE: If reviewer comments were submitted as an attachment file, they will be attached to this email and accessible via the submission site. Please log into your account, locate the manuscript record, and check for the action link "View Attachments". If this link does

not appear, there are no attachment files.]

While revising your submission, please upload your figure files to the Preflight Analysis and Conversion Engine (PACE) digital diagnostic tool, https://pacev2.apexcovantage.com/. PACE helps ensure that figures meet PLOS requirements. To use PACE, you must first register as a user. Registration is free. Then, login and navigate to the UPLOAD tab, where you will find detailed instructions on how to use the tool. If you encounter any issues or have any questions when using PACE, please email PLOS at figures@plos.org. Please note that Supporting Information files do not need this step.
