# Supplementary material for: Initial presentation of early rheumatoid arthritis
Source: PLoS One. 2023 Jul 6;18(7):e0287707. doi: 10.1371/journal.pone.0287707 (PMC10325069; doi:10.1371/journal.pone.0287707)
Supplement: Attachment — Submitted filename: Response_to_reviewers_ March10,2023 .docx [file pone.0287707.s003.docx]

**Lähettäjä:** Lauri Weman <[lauriweman@gmail.com](mailto:lauriweman@gmail.com)>
**Lähetetty:** keskiviikko 25. tammikuuta 2023 18.03
**Vastaanottaja:** Sokka-Isler Tuulikki <[tuulikki.sokka-isler@ksshp.fi](mailto:tuulikki.sokka-isler@ksshp.fi)>
**Aihe:** Fwd: PLOS ONE Decision: Revision required [PONE-D-22-32344] - [EMID:efbd7df372adebd2]

---------- Forwarded message ---------
Lähettäjä: **PLOS ONE** <[em@editorialmanager.com](mailto:em@editorialmanager.com)>
Date: ke 25. tammik. 2023 klo 18.01
Subject: PLOS ONE Decision: Revision required [PONE-D-22-32344] - [EMID:efbd7df372adebd2]
To: Lauri Weman <[lauriweman@gmail.com](mailto:lauriweman@gmail.com)>

CC: [sumar@uic.edu](mailto:sumar@uic.edu), [sadiq36@gmail.com](mailto:sadiq36@gmail.com)

PONE-D-22-32344

Initial presentation of early rheumatoid arthritis

PLOS ONE

Dear Dr. Weman,

Thank you for submitting your manuscript to PLOS ONE. After careful consideration, we feel that it has merit but does not fully meet PLOS ONE’s publication criteria as it currently stands. Therefore, we invite you to submit a revised version of the manuscript that addresses the points raised during the review process.

Please submit your revised manuscript by Mar 11 2023 11:59PM. If you will need more time than this to complete your revisions, please reply to this message or contact the journal office at [plosone@plos.org](mailto:plosone@plos.org). When you're ready to submit your revision, log on to [https://www.editorialmanager.com/pone/](https://eur05.safelinks.protection.outlook.com/?url=https%3A%2F%2Fwww.editorialmanager.com%2Fpone%2F&data=05%7C01%7Ctuulikki.sokka-isler%40ksshp.fi%7Cde59bf8b374c4cf1ae9608dafeedaf5a%7Ce1105d2e0bbf40ae8d8e26a848f42161%7C0%7C0%7C638102594245907699%7CUnknown%7CTWFpbGZsb3d8eyJWIjoiMC4wLjAwMDAiLCJQIjoiV2luMzIiLCJBTiI6Ik1haWwiLCJXVCI6Mn0%3D%7C3000%7C%7C%7C&sdata=Ml9FYW6TtMTqyP01EAUmGPYZxeCuaj%2Fq%2B5kvYBeK%2FZU%3D&reserved=0) and select the 'Submissions Needing Revision' folder to locate your manuscript file.

Please include the following items when submitting your revised manuscript:

- A rebuttal letter that responds to each point raised by the academic editor and reviewer(s). You should upload this letter as a separate file labeled 'Response to Reviewers'.
- A marked-up copy of your manuscript that highlights changes made to the original version. You should upload this as a separate file labeled 'Revised Manuscript with Track Changes'.
- An unmarked version of your revised paper without tracked changes. You should upload this as a separate file labeled 'Manuscript'.

If you would like to make changes to your financial disclosure, please include your updated statement in your cover letter. Guidelines for resubmitting your figure files are available below the reviewer comments at the end of this letter.

If applicable, we recommend that you deposit your laboratory protocols in [protocols.io](https://eur05.safelinks.protection.outlook.com/?url=http%3A%2F%2Fprotocols.io%2F&data=05%7C01%7Ctuulikki.sokka-isler%40ksshp.fi%7Cde59bf8b374c4cf1ae9608dafeedaf5a%7Ce1105d2e0bbf40ae8d8e26a848f42161%7C0%7C0%7C638102594245907699%7CUnknown%7CTWFpbGZsb3d8eyJWIjoiMC4wLjAwMDAiLCJQIjoiV2luMzIiLCJBTiI6Ik1haWwiLCJXVCI6Mn0%3D%7C3000%7C%7C%7C&sdata=oSc03OepmreKdt9TOE01jXJHBim806tdAnNAydJBSMg%3D&reserved=0) to enhance the reproducibility of your results. Protocols.io assigns your protocol its own identifier (DOI) so that it can be cited independently in the future. For instructions see: [https://journals.plos.org/plosone/s/submission-guidelines#loc-laboratory-protocols](https://eur05.safelinks.protection.outlook.com/?url=https%3A%2F%2Fjournals.plos.org%2Fplosone%2Fs%2Fsubmission-guidelines%23loc-laboratory-protocols&data=05%7C01%7Ctuulikki.sokka-isler%40ksshp.fi%7Cde59bf8b374c4cf1ae9608dafeedaf5a%7Ce1105d2e0bbf40ae8d8e26a848f42161%7C0%7C0%7C638102594245907699%7CUnknown%7CTWFpbGZsb3d8eyJWIjoiMC4wLjAwMDAiLCJQIjoiV2luMzIiLCJBTiI6Ik1haWwiLCJXVCI6Mn0%3D%7C3000%7C%7C%7C&sdata=6UFqx1RxdWt51Pfkokm6P9LPxFg7yRWzZAjAbra%2FQNM%3D&reserved=0). Additionally, PLOS ONE offers an option for publishing peer-reviewed Lab Protocol articles, which describe protocols hosted on [protocols.io](https://eur05.safelinks.protection.outlook.com/?url=http%3A%2F%2Fprotocols.io%2F&data=05%7C01%7Ctuulikki.sokka-isler%40ksshp.fi%7Cde59bf8b374c4cf1ae9608dafeedaf5a%7Ce1105d2e0bbf40ae8d8e26a848f42161%7C0%7C0%7C638102594246064443%7CUnknown%7CTWFpbGZsb3d8eyJWIjoiMC4wLjAwMDAiLCJQIjoiV2luMzIiLCJBTiI6Ik1haWwiLCJXVCI6Mn0%3D%7C3000%7C%7C%7C&sdata=9IwmiH2OoujVxDXAlRm66cubq7kUSpoxj4saJsPWiSA%3D&reserved=0). Read more information on sharing protocols at [https://plos.org/protocols?utm_medium=editorial-email&utm_source=authorletters&utm_campaign=protocols](https://eur05.safelinks.protection.outlook.com/?url=https%3A%2F%2Fplos.org%2Fprotocols%3Futm_medium%3Deditorial-email%26utm_source%3Dauthorletters%26utm_campaign%3Dprotocols&data=05%7C01%7Ctuulikki.sokka-isler%40ksshp.fi%7Cde59bf8b374c4cf1ae9608dafeedaf5a%7Ce1105d2e0bbf40ae8d8e26a848f42161%7C0%7C0%7C638102594246064443%7CUnknown%7CTWFpbGZsb3d8eyJWIjoiMC4wLjAwMDAiLCJQIjoiV2luMzIiLCJBTiI6Ik1haWwiLCJXVCI6Mn0%3D%7C3000%7C%7C%7C&sdata=KJt7oAfqCE0bDuChS6l52LupTLs266anGdVQk3Dnof0%3D&reserved=0).

We look forward to receiving your revised manuscript.

Kind regards,

Sadiq Umar
Academic Editor
PLOS ONE

Journal Requirements:

When submitting your revision, we need you to address these additional requirements.

1. Please ensure that your manuscript meets PLOS ONE's style requirements, including those for file naming. The PLOS ONE style templates can be found at 
[https://journals.plos.org/plosone/s/file?id=wjVg/PLOSOne_formatting_sample_main_body.pdf](https://eur05.safelinks.protection.outlook.com/?url=https%3A%2F%2Fjournals.plos.org%2Fplosone%2Fs%2Ffile%3Fid%3DwjVg%2FPLOSOne_formatting_sample_main_body.pdf&data=05%7C01%7Ctuulikki.sokka-isler%40ksshp.fi%7Cde59bf8b374c4cf1ae9608dafeedaf5a%7Ce1105d2e0bbf40ae8d8e26a848f42161%7C0%7C0%7C638102594246064443%7CUnknown%7CTWFpbGZsb3d8eyJWIjoiMC4wLjAwMDAiLCJQIjoiV2luMzIiLCJBTiI6Ik1haWwiLCJXVCI6Mn0%3D%7C3000%7C%7C%7C&sdata=R21%2BpvG1MBy7FVLdwTTJc6gJih5LvpwRFTsTn7twP9w%3D&reserved=0) and 
[https://journals.plos.org/plosone/s/file?id=ba62/PLOSOne_formatting_sample_title_authors_affiliations.pdf](https://eur05.safelinks.protection.outlook.com/?url=https%3A%2F%2Fjournals.plos.org%2Fplosone%2Fs%2Ffile%3Fid%3Dba62%2FPLOSOne_formatting_sample_title_authors_affiliations.pdf&data=05%7C01%7Ctuulikki.sokka-isler%40ksshp.fi%7Cde59bf8b374c4cf1ae9608dafeedaf5a%7Ce1105d2e0bbf40ae8d8e26a848f42161%7C0%7C0%7C638102594246064443%7CUnknown%7CTWFpbGZsb3d8eyJWIjoiMC4wLjAwMDAiLCJQIjoiV2luMzIiLCJBTiI6Ik1haWwiLCJXVCI6Mn0%3D%7C3000%7C%7C%7C&sdata=xPxRlPY2rA%2FNF482JM5fUpMSOvDM%2BFKwVa5oCK7MipE%3D&reserved=0)

2. We note that the grant information you provided in the ‘Funding Information’ and ‘Financial Disclosure’ sections do not match.

When you resubmit, please ensure that you provide the correct grant numbers for the awards you received for your study in the ‘Funding Information’ section.

3. Thank you for stating the following in the Acknowledgments Section of your manuscript: 
   "We would like to thank The Finnish Psoriasis Association, The Finnish Society for Rheumatology,and Amgen. The author also acknowledges the Research Committee of the Kuopio University Hospital Catchment Area for the State Research Funding (project 5041730, Kuopio, Finland)."

We note that you have provided funding information that is not currently declared in your Funding Statement. However, funding information should not appear in the Acknowledgments section or other areas of your manuscript. We will only publish funding information present in the Funding Statement section of the online submission form. 
Please remove any funding-related text from the manuscript and let us know how you would like to update your Funding Statement. Currently, your Funding Statement reads as follows: 
  "NO"
 
Please include your amended statements within your cover letter; we will change the online submission form on your behalf.

4. Thank you for stating the following in your Competing Interests section:  
   "NO"
Please complete your Competing Interests on the online submission form to state any Competing Interests. If you have no competing interests, please state "The authors have declared that no competing interests exist.", as detailed online in our guide for authors at [http://journals.plos.org/plosone/s/submit-now](https://eur05.safelinks.protection.outlook.com/?url=http%3A%2F%2Fjournals.plos.org%2Fplosone%2Fs%2Fsubmit-now&data=05%7C01%7Ctuulikki.sokka-isler%40ksshp.fi%7Cde59bf8b374c4cf1ae9608dafeedaf5a%7Ce1105d2e0bbf40ae8d8e26a848f42161%7C0%7C0%7C638102594246064443%7CUnknown%7CTWFpbGZsb3d8eyJWIjoiMC4wLjAwMDAiLCJQIjoiV2luMzIiLCJBTiI6Ik1haWwiLCJXVCI6Mn0%3D%7C3000%7C%7C%7C&sdata=Gv2OKLCQ3Xk9ntGNczk620W5QgOziNHjJmJwe3zJMiw%3D&reserved=0) 
 This information should be included in your cover letter; we will change the online submission form on your behalf.

5. In your Data Availability statement, you have not specified where the minimal data set underlying the results described in your manuscript can be found. PLOS defines a study's minimal data set as the underlying data used to reach the conclusions drawn in the manuscript and any additional data required to replicate the reported study findings in their entirety. All PLOS journals require that the minimal data set be made fully available. For more information about our data policy, please see [http://journals.plos.org/plosone/s/data-availability](https://eur05.safelinks.protection.outlook.com/?url=http%3A%2F%2Fjournals.plos.org%2Fplosone%2Fs%2Fdata-availability&data=05%7C01%7Ctuulikki.sokka-isler%40ksshp.fi%7Cde59bf8b374c4cf1ae9608dafeedaf5a%7Ce1105d2e0bbf40ae8d8e26a848f42161%7C0%7C0%7C638102594246064443%7CUnknown%7CTWFpbGZsb3d8eyJWIjoiMC4wLjAwMDAiLCJQIjoiV2luMzIiLCJBTiI6Ik1haWwiLCJXVCI6Mn0%3D%7C3000%7C%7C%7C&sdata=kw%2BVwNFU%2FYqbCEgF1xbHKnc8StGlgwWx%2Biny0cYY%2F6k%3D&reserved=0).

"Upon re-submitting your revised manuscript, please upload your study’s minimal underlying data set as either Supporting Information files or to a stable, public repository and include the relevant URLs, DOIs, or accession numbers within your revised cover letter. For a list of acceptable repositories, please see [http://journals.plos.org/plosone/s/data-availability#loc-recommended-repositories](https://eur05.safelinks.protection.outlook.com/?url=http%3A%2F%2Fjournals.plos.org%2Fplosone%2Fs%2Fdata-availability%23loc-recommended-repositories&data=05%7C01%7Ctuulikki.sokka-isler%40ksshp.fi%7Cde59bf8b374c4cf1ae9608dafeedaf5a%7Ce1105d2e0bbf40ae8d8e26a848f42161%7C0%7C0%7C638102594246064443%7CUnknown%7CTWFpbGZsb3d8eyJWIjoiMC4wLjAwMDAiLCJQIjoiV2luMzIiLCJBTiI6Ik1haWwiLCJXVCI6Mn0%3D%7C3000%7C%7C%7C&sdata=xB0UtX8MaAblbYRl8xdXwtsG8i1QSgZExhvBrZ8GaB4%3D&reserved=0). Any potentially identifying patient information must be fully anonymized.

Important: If there are ethical or legal restrictions to sharing your data publicly, please explain these restrictions in detail. Please see our guidelines for more information on what we consider unacceptable restrictions to publicly sharing data: [http://journals.plos.org/plosone/s/data-availability#loc-unacceptable-data-access-restrictions](https://eur05.safelinks.protection.outlook.com/?url=http%3A%2F%2Fjournals.plos.org%2Fplosone%2Fs%2Fdata-availability%23loc-unacceptable-data-access-restrictions&data=05%7C01%7Ctuulikki.sokka-isler%40ksshp.fi%7Cde59bf8b374c4cf1ae9608dafeedaf5a%7Ce1105d2e0bbf40ae8d8e26a848f42161%7C0%7C0%7C638102594246064443%7CUnknown%7CTWFpbGZsb3d8eyJWIjoiMC4wLjAwMDAiLCJQIjoiV2luMzIiLCJBTiI6Ik1haWwiLCJXVCI6Mn0%3D%7C3000%7C%7C%7C&sdata=oGelg8NPInllC4GfJVj7GFyeSI2CRBeLLCbvOwNGHHw%3D&reserved=0). Note that it is not acceptable for the authors to be the sole named individuals responsible for ensuring data access.

We will update your Data Availability statement to reflect the information you provide in your cover letter.

6. PLOS requires an ORCID iD for the corresponding author in Editorial Manager on papers submitted after December 6th, 2016. Please ensure that you have an ORCID iD and that it is validated in Editorial Manager. To do this, go to ‘Update my Information’ (in the upper left-hand corner of the main menu), and click on the Fetch/Validate link next to the ORCID field. This will take you to the ORCID site and allow you to create a new iD or authenticate a pre-existing iD in Editorial Manager. Please see the following video for instructions on linking an ORCID iD to your Editorial Manager account: [https://www.youtube.com/watch?v=_xcclfuvtxQ](https://eur05.safelinks.protection.outlook.com/?url=https%3A%2F%2Fwww.youtube.com%2Fwatch%3Fv%3D_xcclfuvtxQ&data=05%7C01%7Ctuulikki.sokka-isler%40ksshp.fi%7Cde59bf8b374c4cf1ae9608dafeedaf5a%7Ce1105d2e0bbf40ae8d8e26a848f42161%7C0%7C0%7C638102594246064443%7CUnknown%7CTWFpbGZsb3d8eyJWIjoiMC4wLjAwMDAiLCJQIjoiV2luMzIiLCJBTiI6Ik1haWwiLCJXVCI6Mn0%3D%7C3000%7C%7C%7C&sdata=9ji2rf8qrZWxRP0x4l913lBCx2sB8xPT6AI%2FFrjp4%2Fg%3D&reserved=0)

7. Please ensure that you include a title page within your main document. You should list all authors and all affiliations as per our author instructions and clearly indicate the corresponding author.

8. We note that you have included the phrase “data not shown” in your manuscript. Unfortunately, this does not meet our data sharing requirements. PLOS does not permit references to inaccessible data. We require that authors provide all relevant data within the paper, Supporting Information files, or in an acceptable, public repository. Please add a citation to support this phrase or upload the data that corresponds with these findings to a stable repository (such as Figshare or Dryad) and provide and URLs, DOIs, or accession numbers that may be used to access these data. Or, if the data are not a core part of the research being presented in your study, we ask that you remove the phrase that refers to these data.

9. Please include your full ethics statement in the ‘Methods’ section of your manuscript file. In your statement, please include the full name of the IRB or ethics committee who approved or waived your study, as well as whether or not you obtained informed written or verbal consent. If consent was waived for your study, please include this information in your statement as well.

10. Please include a copy of Table 3 and 4 which you refer to in your text on page 10 and 15.

11. Please include captions for your Supporting Information files at the end of your manuscript, and update any in-text citations to match accordingly. Please see our Supporting Information guidelines for more information: [http://journals.plos.org/plosone/s/supporting-information](https://eur05.safelinks.protection.outlook.com/?url=http%3A%2F%2Fjournals.plos.org%2Fplosone%2Fs%2Fsupporting-information&data=05%7C01%7Ctuulikki.sokka-isler%40ksshp.fi%7Cde59bf8b374c4cf1ae9608dafeedaf5a%7Ce1105d2e0bbf40ae8d8e26a848f42161%7C0%7C0%7C638102594246064443%7CUnknown%7CTWFpbGZsb3d8eyJWIjoiMC4wLjAwMDAiLCJQIjoiV2luMzIiLCJBTiI6Ik1haWwiLCJXVCI6Mn0%3D%7C3000%7C%7C%7C&sdata=g3zSZJGgPkQ6Zgt42dPQZEn3z84nYScTuZ1KlSwzjYA%3D&reserved=0).

[Note: HTML markup is below. Please do not edit.]

Reviewers' comments:

Reviewer's Responses to Questions

**Comments to the Author**

1. Is the manuscript technically sound, and do the data support the conclusions?

The manuscript must describe a technically sound piece of scientific research with data that supports the conclusions. Experiments must have been conducted rigorously, with appropriate controls, replication, and sample sizes. The conclusions must be drawn appropriately based on the data presented.

Reviewer #1: Yes

Reviewer #2: Yes

2. Has the statistical analysis been performed appropriately and rigorously?

Reviewer #1: Yes

Reviewer #2: Yes

3. Have the authors made all data underlying the findings in their manuscript fully available?

The [PLOS Data policy](https://eur05.safelinks.protection.outlook.com/?url=http%3A%2F%2Fwww.plosone.org%2Fstatic%2Fpolicies.action%23sharing&data=05%7C01%7Ctuulikki.sokka-isler%40ksshp.fi%7Cde59bf8b374c4cf1ae9608dafeedaf5a%7Ce1105d2e0bbf40ae8d8e26a848f42161%7C0%7C0%7C638102594246064443%7CUnknown%7CTWFpbGZsb3d8eyJWIjoiMC4wLjAwMDAiLCJQIjoiV2luMzIiLCJBTiI6Ik1haWwiLCJXVCI6Mn0%3D%7C3000%7C%7C%7C&sdata=oCqEet6tT2zSEzSnuhHqNYf%2F74pc0LbDufo1GvtlIZw%3D&reserved=0) requires authors to make all data underlying the findings described in their manuscript fully available without restriction, with rare exception (please refer to the Data Availability Statement in the manuscript PDF file). The data should be provided as part of the manuscript or its supporting information, or deposited to a public repository. For example, in addition to summary statistics, the data points behind means, medians and variance measures should be available. If there are restrictions on publicly sharing data—e.g. participant privacy or use of data from a third party—those must be specified.

Reviewer #1: Yes

Reviewer #2: No

4. Is the manuscript presented in an intelligible fashion and written in standard English?

PLOS ONE does not copyedit accepted manuscripts, so the language in submitted articles must be clear, correct, and unambiguous. Any typographical or grammatical errors should be corrected at revision, so please note any specific errors here.

Reviewer #1: Yes

Reviewer #2: Yes

5. Review Comments to the Author

Please use the space provided to explain your answers to the questions above. You may also include additional comments for the author, including concerns about dual publication, research ethics, or publication ethics. (Please upload your review as an attachment if it exceeds 20,000 characters)

**Reviewer #1:** The authors describe the initial presentation of early rheumatoid arthritis patients in Finland, using national databases. The result may be of interesting to readers, however, I have a couple of concerns. Specific comments are listed below:

1. The authors compare clinical items between ACPA-positive RA patients and negative patients, this is a bit confusing because so called seropositive RA includes RF+/ACPA- patients. This study may include such patients in ACPA negative RA patients, which shows a bit different nuance compared to 'seronegative' RA. More explanation of this may be needed to clarify the characteristics of patient group. I also be interested the clinical feature of ACPA-/RF+ RA patients (can be difficult to investigate, though).

**Response:** According to the Reviewers’s suggestion, we now present the data for seropositive (ACPA or RF positive) vs. seronegative (double negative) patients. In addition, we present a separate table for 163 ACPA+/RF- patients vs. 118 ACPA-/RF+ patients.

2. We routinely use EULAR/ACR criteria for RA diagnosis in daily clinical practice, and due to the setting of scoring system, it is understandable ACPA negative (seronegative??) RA patients show more swollen joints (symmetrical) or high CRP levels. Therefore, it is natural to predict the results in this study. In other words, what is the most interesting findings in this study, compared to previous findings? Please address more clearly.

**Response:** We have shown (Weman&Sokka, Duodecim 2021, in Finnish) in an analysis from our own clinic, that RA does not present as polyarthritis but the initial presentation currently is arthritis in 1-2 joints in more than 50% of patients + positive ACPA. This is what he hypothesized concerning patients from the whole country. Unfortunately this is not true and we have to report that in Finland, the initial presentation of RA is still symmetric polyarthritis. - However, our group felt that we cannot open this up in the manuscript but merely report the findings.

3. This study dose not describe initial therapy after diagnosis of RA. Are there any differences (if can)?

**Response:** We added the analyses of the initial medication.

**Reviewer #2**: The authors studied the initial clinical presentation of alarge number of RA patients using national databases from Finland, with a particular interest in the impact of the presence of ACPA on the clinical presentation. A fair concern related to the objective of this study is that patients that do not present with symmetric polyarthritis could be having their diagnosis delayed. They concluded that incident RA presents mainly as symmetric arthritis, with higher disease severity in ACPA-negative patients. . This is not surprising, since as the authors themselves mentioned, the current classification criteria gives weight to seropositivity and, in negative patients, to the number of joints. On the other hand, it was surprising that ACAP-pos patients had a longer delay in diagnosis than ACPA-neg, when we know that ACPA is correlated with disease severity. For me, this suggests that there may be present a recruitment bias in this study: the ACPA-neg patients recruited could be representative of a more severe spectrum, while patients with milder forms are being left undiagnosed as RA, perhaps receiving other labels, such as "undifferentiated arthritis". The authors should discuss that possibility since it can have a great bearing on the main conclusions of the study. 

**Response:** This result that ACPA positive patients had a longer delay in diagnosis vs. ACPA negative was also surprising to us. It is possible that the double seronegative group included patients who should have been labelled as polymyalgia rheumatica. However through this database we cannot investigate this further. We included this in discussion.

Other issues/recommendations: 

1. The authors should comment on the criteria for the request of ACPA, since about 11% of incident RA patients were not included in the analysis because they did not have ACPA available in their records. Could all these patients be RF positive, with high disease activity and, therefore, ACPA was forsaken because of obvious diagnosis? This could alter some of the conclusions that ACPA-neg patients have higher disease activity and shorter diagnostic delay.

**Response**: Practically all patients with the suspicion of RA are being tested for ACPA and RF in Finland. The reason for the missing ACPA/RF values is simple: there was a delay in the programming of the automatic data transfer from the laboratory databases and therefore, the ACPA/RF values had to available from the clinical databases where the values had to be entered by hand. If the “hand work” was missing, the values were missing in these analyses. However, in the future, all laboratory data taken will be available in the national rheumatology database, as soon as the automatic laboratory transfer will be ready.

The reviewer asks whether all these patients who had missing ACPA were RF positive and therefore ACPA would not even had been taken – and had high disease activity. This was not the case. The 216 patients that weren’t included in the analyses had a median duration of symptoms of 6 months, being longer compared to the patients who were ACPA-tested. The median DAS28-score was 3.6 for these 216 patients, whereas the median DAS28 was 3.7 for ACPA-positive patients and 4.5 for ACPA-negative patients.

2. The variables in Methods should be presented in full sentences, with better descriptions, and not by hyphenated-items. Particular confusing are the variables used to represent the duration of disease: "duration of symptoms", "fulfillment of ACR/EULAR 2010-criteria" and "diagnostic delay". These terms should be standardized throughout the text.

**Response:** We have now explained the two variables with more detail. The time between first symptoms and the diagnosis of RA is now referred only as ‘’duration of symptoms’’.

-duration of symptoms: time between the first symptoms of RA and the diagnosis of RA

-Fulfillment of the ACR/EULAR 2010 criteria: clinical diagnosis of RA by rheumatologist who counts the items in the ACR/EULAR 2010 criteria, if score adds up to 6 or more points, the ACR/EULAR 2010 criteria are met, when also other reasons that can explain the condition are excluded.

3. In terms of disease duration, the median (IQR) of 4 (2,10) months is very short, indicating that most (if not all) patients have relatively early (< 1y) disease. This should be discussed: is it true that very few patients in Finland are being diagnosed with longer than 1 year disease duration?  Perhaps the ranges of disease duration could be presented, particularly for the groups by diagnosis delay.

**Response:** We present the range of disease duration in S2 table.

Concerning the “short” duration of symptoms before diagnosis we want to explain the setting of the Finnish rheumatology care:

Historically, there has been a strong vision to get RA under control in Finland. The Rheumatism Foundation Hospital was established in 1951 in Heinola and it also served as the national center of research and education in rheumatic diseases. Their vision was well described in a letter published in Br Med Journal in 1978, as a response to critique to their enthusiasm concerning intra muscular gold in early RA: “Gold treatment ought to be started before erosions…. We are treating not only the actual inflammation of the joints but also the quality of the patient’s life for many decades in the future”. (Luukkainen R, Kajander A, Isomäki H. Br Med J 1978; 2:1501). This was the Finnish manifest of T2T, 30 years before its time. The FIN-RAco study in the 1990’s increased the awareness of the urgency to make the diagnosis of RA early. The availability of the ACPA test in early 2000’s helped to recognize true seropositive cases of RA.

Education. There are only five medical schools in Finland; rheumatology educators are a small team and it has been clear for long that every medical student has to know, how to suspect early RA, test ACPA, and make a referral. Furthermore, national “general practice guidelines” are available in the internet (since 20 or so years, and updated regularly) to all health care professionals, e.g. what to do if a patient has a swollen joint – or a suspicion of a swollen joint.

Structure of health care is based on shared care between general health care and specialty care. Diagnosis of RA belongs to the rheumatology multidisciplinary clinic, with specialized nurses for patient education. The “early RA treatment path” describes the follow-up of the patient and the role of general health care and the rheumatology clinic.

Therefore, it is not surprising to us that the median duration of symptoms before diagnosis was 4 months nationwide. In fact, it should be shorter as we discuss in the manuscript.

4. Tables 3 and 4 are supplementary - this should be indicated in the main text when they are referred to.

**Response:**They are now referred as S1 table and S2 table, according to the manuscript guidelines.

6. PLOS authors have the option to publish the peer review history of their article ([what does this mean?](https://eur05.safelinks.protection.outlook.com/?url=https%3A%2F%2Fjournals.plos.org%2Fplosone%2Fs%2Feditorial-and-peer-review-process%23loc-peer-review-history&data=05%7C01%7Ctuulikki.sokka-isler%40ksshp.fi%7Cde59bf8b374c4cf1ae9608dafeedaf5a%7Ce1105d2e0bbf40ae8d8e26a848f42161%7C0%7C0%7C638102594246064443%7CUnknown%7CTWFpbGZsb3d8eyJWIjoiMC4wLjAwMDAiLCJQIjoiV2luMzIiLCJBTiI6Ik1haWwiLCJXVCI6Mn0%3D%7C3000%7C%7C%7C&sdata=OnFHki0D5o6idAnIU%2BGdC1Wi7b%2BONg2XtKoIoNCap70%3D&reserved=0)). If published, this will include your full peer review and any attached files.

If you choose “no”, your identity will remain anonymous but your review may still be made public.

**Do you want your identity to be public for this peer review?** For information about this choice, including consent withdrawal, please see our [Privacy Policy](https://eur05.safelinks.protection.outlook.com/?url=https%3A%2F%2Fwww.plos.org%2Fprivacy-policy&data=05%7C01%7Ctuulikki.sokka-isler%40ksshp.fi%7Cde59bf8b374c4cf1ae9608dafeedaf5a%7Ce1105d2e0bbf40ae8d8e26a848f42161%7C0%7C0%7C638102594246064443%7CUnknown%7CTWFpbGZsb3d8eyJWIjoiMC4wLjAwMDAiLCJQIjoiV2luMzIiLCJBTiI6Ik1haWwiLCJXVCI6Mn0%3D%7C3000%7C%7C%7C&sdata=bRVlvcQE%2BvDAiZL2jFYTviYqDbqPJ%2Ft32kSdh4L4abI%3D&reserved=0).

Reviewer #1: No

Reviewer #2: No

[NOTE: If reviewer comments were submitted as an attachment file, they will be attached to this email and accessible via the submission site. Please log into your account, locate the manuscript record, and check for the action link "View Attachments". If this link does not appear, there are no attachment files.]

While revising your submission, please upload your figure files to the Preflight Analysis and Conversion Engine (PACE) digital diagnostic tool, [https://pacev2.apexcovantage.com/](https://eur05.safelinks.protection.outlook.com/?url=https%3A%2F%2Fpacev2.apexcovantage.com%2F&data=05%7C01%7Ctuulikki.sokka-isler%40ksshp.fi%7Cde59bf8b374c4cf1ae9608dafeedaf5a%7Ce1105d2e0bbf40ae8d8e26a848f42161%7C0%7C0%7C638102594246064443%7CUnknown%7CTWFpbGZsb3d8eyJWIjoiMC4wLjAwMDAiLCJQIjoiV2luMzIiLCJBTiI6Ik1haWwiLCJXVCI6Mn0%3D%7C3000%7C%7C%7C&sdata=Raq9a%2Bhoj7Gd05oJ2dNmGwIDizn8ipzm0IthimHZUJ4%3D&reserved=0). PACE helps ensure that figures meet PLOS requirements. To use PACE, you must first register as a user. Registration is free. Then, login and navigate to the UPLOAD tab, where you will find detailed instructions on how to use the tool. If you encounter any issues or have any questions when using PACE, please email PLOS at [figures@plos.org](mailto:figures@plos.org). Please note that Supporting Information files do not need this step.
